# European Musculo-Skeletal Oncology Society Meeting, 24 & 25 May 2001, Pamplona: Abstracts and Poster Presentation and Nurses Symposium

**DOI:** 10.1080/13577140120099236

**Published:** 2001-12


*Sarcoma* (2001) 5, 219–243

### European Musculo-Skeletal Oncology Society Meeting, 24 & 25 May 2001, Pamplona: Abstracts and Poster Presentation and Nurses Symposium

### ORAL PRESENTATIONS

### Session 1: Prognostic and therapeutic applications of molecular genetics in musculo-skeletal tumours

**Sarcoma risk, disease severity and EXT mutation in hereditary multiple exostoses: a genotype-phenotype study**

D.E. PORTER[1], M. FRASER[1], C. DOBSON-STONE[2], L. LONIE[2], A.M. MONACO[2], & A.H.R.W. SIMPSON[1]
*([1]Nuffield Department of Orthopaedic Surgery, Nuffield Orthopaedic Centre, [2]Wellcome Trust Centre for Human Genetics, Headington, Oxford, UK)*

*Introduction and Aims:* Hereditary multiple exostoses (HME) is the commonest autosomal dominant orthopaedic neoplastic condition, affecting 1/50 000. Recent studies show that EXT1 (chromo. 8q) and EXT2 (chromo. 11p) are responsible for about 80% of families. Molecular studies suggest the EXT1 (but perhaps not EXT2) gene is important in progression of osteochondroma to chondrosarcoma. In the only study of its kind, we aimed to determine whether disease severity (and sarcoma risk) is worse in EXT1 than EXT2 mutation carriers.
*Methods:* From 1996 to 2000, 172 affected individuals from 78 families with HME were screened for EXT mutation using fluorescent single-strand conformational polymorphism (f-SSCP) and DNA sequence analysis. Prior to mutation screening, a structured history and clinical examination by two examiners allowed an HME score to be calculated based on anatomical, functional and surgical criteria (number of palpable osteochondromas, forearm and lower leg deformity, radial head dislocation, range of movement at elbow, forearm, wrist, knee, centile height, ulnar length as a proportion of height, previous surgery). Statistical analysis was performed using non-parametric (chi-square, Wilcoxon, Kendall's)tests.
*Results:* 72 EXT1 and 70 EXT2 individuals were identified (no mutation in 30). HME score ranged from 4 to 81 out of 100, and was age- and sex-independent. Median HME score was significantly greater in EXT1 than EXT2 carriers (median scores 44 vs 35, $P<0.001$). When broken down into anatomical, functional and surgical components, each remained significantly worse in EXT1 carriers. Six sarcomas occurred, all in EXT1 carriers. Those with sarcoma did not have significantly worse disease than other EXT1 mutation carriers.
*Discussion:* For the first time, EXT1 has been shown to produce worse disease than EXT2 in HME. A greater risk of sarcoma may result either from larger number of osteochondromas or because EXT1 mutation is more crucial than EXT2 in the stepwise tumorigenic process. The identification of a higher-risk group (EXT1) within the HME cohort may allow a better rationale for targetted screening to seek worrying lesions prior to malignant change

**Chromosome 9 alterations and p16 protein expression in central chondrosarcomas**

JUDITH V.M.G. BOVÉE[1], DMITRI FEDOROV[1], ANTHONIE H.M. TAMINIAU[2], RAF SCIOT[3], MARIA DEBIEC-RYCHTER[4], ANNE-MARIE CLETON-JANSEN[1] & PANCRAS C.W. HOGENDOORN[1]
*(Departments of [1]Pathology and [2]Orthopaedic Surgery, Leiden University Medical Center, Leiden, The Netherlands; [3]Department of Pathology and [4]Center for Human Genetics, Leuven University, Leuven, Belgium)*

*Aim:* Chondrosarcomas are characterized by neoplastic growth of cartilage forming tumor cells. The majority (75%) arise centrally, in the medullar cavity, while a minority develop peripherally secondary to an osteochondroma. We previously investigated DNA-ploidy and loss of heterozygosity (LOH) at loci harboring the *EXT*-genes (implicated in hereditary multiple exostoses), the *EXT*-like genes, and at 9p21, 13q14, 17p13 and chromosome 10 in 12 central chondrosarcomas. Only three cases exhibited LOH, with 9p21 involved in all three. At 9p21 the p16 tumour suppressor gene is located. Our goal was to further investigate this chromosomal region and the expression of the candidate gene p16.
*Methods:* Cytogenetic analysis was performed on 16 central chondrosarcomas. p16 immunohistochemistry was performed on formalin-fixed paraffin-embedded tissue of 16 cases to estimate the effect of 9p21 alterations on p16 protein expression.
*Results:* Seven central chondrosarcomas demonstrated an abnormal karyotype, five of which involved chromosome 9. Three central tumours showed involvement of the 9p12–22 region including t(9;10)(p22;q22), add(9)(p21) and add(9)(p12). For two of them paraffin blocks were available revealing absence of p16 protein expression. Two tumours with –9 and del(9)(q12) demonstrated p16 protein expression. In three chondrosarcomas previously demonstrating LOH at 9p21, p16 protein expression was absent, while in six out of nine central chondrosarcomas without LOH at 9p21, p16 protein expression could be demonstrated.
*Conclusions:* The involvement of genes located at chromosome 9, especially the 9p12–22 region, is suggested both by the previous LOH results as well as the present cytogenetic studies. Since 9p21 alterations are highly associated with the absence of p16 protein expression, this suggests an important role for the p16 tumour suppressor gene in the development of central chondrosarcomas.

**Relevance of c-Kit as a prognostic marker and therapeutic target in Ewing's sarcoma**

K. SCOTLANDI, R. STRAMMIELLO, M.C. MANARA, V. CERISANO, S. BENINI, M. SERRA, P. NANNI*, L. LANDUZZI[§] & P. PICCI
*(Laboratorio di Ricerca Oncologica, Istituti Ortopedici Rizzoli, [§]I.S.T., Istituto di Ricerche per la Ricerca sul Cancro, Unità Satellite di Biotecnologie di Bologna, and *Isituto di Cancerologia, Università degli Studi di Bologna, Bologna, Italy)*

1357–714X print/1369–1643 online/01/040219–25 © 2001 Taylor & Francis Ltd
DOI: 10.1080/13577140120099236

Ewing's sarcoma (ES), the second most frequent primary tumor of bone, shows a high tendency to give pulmonary and bone metastases. In particular, skeletal involvement may appear, at the onset or during the course of the disease, also in the absence of lung metastases. The pathogenesis of these lesions, however, is still poorly understood due to the lack of appropriate models. We described the ability of TC-71 and 6647 ES cells to metastasize to the lungs and bones after intravenous injection of tumor cells in nude mice. Moreover, recent findings have indicated in *c-Kit* a possible 'homing' receptor for ES cells. Differently from other musculoskeletal tumors, such as osteosarcoma and rhabdomyosarcoma, ES cell lines showed high expression levels of *c-Kit* receptor. Its ligand SCF, which is produced by human bone marrow stromal cells and endothelial cells, confer a growth advantage to ES cells *in vitro*, due to an increase in cell proliferation and to a reduction in the apoptotic rate, and act as a potent chemoattractant for ES cells. Down-modulation of *c-Kit* expression leads to a reduced chemotactic and metastatic ability of ES cells. In this study, we analyzed the prognostic relevance of the expression of *c-Kit* in a in a consecutive series of patients with ES of the extremities. The expression of *c-Kit* in tumor specimens was assessed by immunohistochemistry in 34 non-metastatic, operable ES treated at a single institution. The *c-Kit* expression was analyzed in relation to event-free survival. *c-Kit* was found to be expressed in 14/34 (42%) of the cases. A widespread pattern of *c-Kit* expression in tumor cells at diagnosis was significantly associated with a higher rate of systemic relapse ($P$=0.02). These findings support the view of *c-Kit* as a promising prognostic marker and therapeutic target in ES.

**Genetic imbalances revealed by comparative genomic hybridization in Ewing tumors**

TOSHIFUMI OZAKI[1], MICHAEL PAULUSSEN[2], CHRISTOPHER POREMBA[3], CHRISTIAN BRINKSCHMIDT[3], JULIO RERIN[2], SUSANNE AHRENS[2], CHRISTIANE HOFFMANN[1], AXEL HILLMANN[1], NORBERT LINDNER[1], DANIEL WAI[3], KARL-LUDWIG SCHÄFER[3], WERNER BÖCKER[3], HERBERT JÜRGENS[2], WINFRIED WINKELMANN[1] & BARBARA DOCKHORN-DWORNICZAK[3]
*(Department of [1]Orthopaedics, [2]Pediatric Hematology and Oncology, and [3]Gerhard-Domagk-Institute of Pathology, Westfälische Wilhelms-University, Münster, Germany)*

Ewing tumors are characterized by reciprocal translocations involving the *EWS* gene on 22q12 fused to ETS transcription-factor family members. Little is known about further aberrations contributing to tumor development and progression.

Sixty-two frozen tumors with known *EWS* rearrangements (52 primary tumors, 10 relapses) of ET patients registered in the EICESS protocol were analyzed by comparative genomic hybridization.

Average number of changes in 52 primary cases and 10 relapsed cases was 3.6 and 4.7 per tumor ($P$=0.153). Frequent abnormalities included gains of chromosomes 8, 12, 20, and 1q and losses of 16q and 19q. Neither number nor type of aberration was associated with histology, tumor size, disease stage, tumor localization, or histological tumor response to chemotherapy. In 52 primary tumors, 26 tumors with type I fusion (*EWS* exon 7 to *FLI1* exon 6) and 26 tumors with other fusion types had an average of 2.4 and 4.8 aberrations per tumor, respectively ($P$=0.031). Combinations of gains of chromosomes 8 and 12, gains of chromosome 20 and either gains of 8q or 18q, and losses of 16q and 17p frequently occurred. The cumulative overall survival (OAS) was different between 35 patients with <5 aberrations and 13 patients with ≥5 aberrations ($P$=0.009). Univariate analysis showed patients with gains of 1q, 2q, 12, and 20 or losses of 16q and 17p had significantly lower OAS than those without aberrations. By multivariate analysis, loss of 16q (relative risk [RR]=0.190; $P$=0.0006) was an independent prognostic factor.

In the development of ETs, the first step is likely to be a fusion between the *EWS* gene and genes encoding members of the ETS family of transcription factors. The chromosomal imbalances in ETs are most likely secondary events involved in tumor progression.

**uPA, uPAR and PAI1 protein expression in giant cell tumors**

M.S. BENASSI, G. GAMBERI, P. RAGAZZINI, G. MAGAGNOLI, L. PAZZAGLIA, F. PONTICELLI, C. FERRARI & P. PICCI
*(Laboratory of Cancer Research, Rizzoli Orthopaedic Institute, Bologna, Italy)*

*Study:* Giant cell tumor (GCT) is a bone neoplasm which is characterized by the presence of large numbers of multinucleated osteoclast-like giant cells. Although GCT can be considered a benign lesion, it exhibits high aggressiveness often associated with osteolytic properties. The urokinase-type plasminogen activation system has been described to be frequently implicated in the process of degradation of the extracellular matrix during tumor cell proliferation and metastasis. The system includes plasmin, its precursor plasminogen, the urokinase-type plasminogen activator (u-PA), and the plasminogen activator inhibitor type 1 (PAI1), which modulates the catalytic action of uPA. Moreover the action of the plasminogen activators is facilitated by the presence of receptors for u-PA on cell surfaces (uPAR).

*Aim;* in this study we examined the expression of the uPA, uPAR and PAI1 proteins in a series of 50 primary GCT and in eight lung metastases, to evaluate if these proteins are associated with bone distruption and metastasis in this form of cancer.

*Method:* the protein expression was studied with the avidin–biotin immunoistochemistry method.

*Results:* our results showed that the incidence of the uPA, uPAR, PAI1 protein overexpression was significantly higher in the relapsed compared to the disease-free patients.

*Conclusion:* these results suggest that overexpression of these proteins could be an additional factor required for the development of invasion in the giant cell tumors.

M. Campanacci *et al.* Tumori 1989; 75: 389–95.
P.A. Andreasen *et al. Int J Cancer* 1997; 72: 1–22.

**Proliferation versus apoptosis in Langerhans' cell histiocytosis**

B. SCHOUTEN[1], R.M. EGELER[1*], P.J.M. LEENEN[2], A.H.M. TAMINIAU[1] & P.C.W. HOGENDOORN[1]
*([1]Department of Pathology, Pediatrics and Orthopedics, Leiden University Medical Center, [2]Department of Immunology, Erasmus University Rotterdam, The Netherlands)*

Langerhans cell histiocytosis (LCH) is characterised by an abnormal proliferation of clonal dendritic cells; the Langerhans cell. These lesions may present amongst others in bones but other sites such as skin or lung might be involved as well. On the basis of clonality of the CD1a+ cell and the reports of familiar clustering in LCH the hypothesis was made that a genetic mutation on the cellular level might be causative for LCH.

*Objectives of the study:* Both osseous as well as skin cd1a+ lesions of 30 patients with LCH were studied with immunohistochemistry for the expression of cell cycle-related gene products p53, mdm2, p16, p21, rb and bcl2.

*Results:* Twenty-seven of 30 cases studied showed staining of the nucleus for p53. The overexpression was heterogeneous. All 27 cases showed strong staining of the nucleus for p21. Both the Langerhans cell showed staining as scattered multinucleated osteoclastic giant cells. All but cases (27) showed strong, diffuse staining of the nucleus and the cytoplasm for p16. Twenty-eight cases were positive for staining with mdm2. the overexpression was heterogeneous, ranging from small focal to strong diffuse staining. All but one of the lesions stained positive for bcl2 ranging from focal small staining in some cases to diffuse strong staining in others. the staining was mostly cytoplasmic.

*Conclusion:* These findings suggest that the cellular mechanisms that sense DNA damage, namely the p53–p21 pathway and the p16–Rb pathway are active and are inducing either cell cycle progression and/or apoptosis. The overexpression of bcl2 might be responsible for the arrest of apoptosis.

**Analysis and prognostic value of the clinical and molecular variables in a series of pediatric osteosarcomas**

A. PATI O GARCÍA, E. SOTILLO PI EIRO, M. ZALACAIN DÍEZ, L. GARATE ITURRIAGAGOITIA,* M. SAN JULIÁN* & L. SIERRASESÚMAGA

*(Department of Pediatrics and *Orthopaedics, University of Navarra, Pamplona, Spain)*

Paediatric osteosarcoma (OS) is well characterised at the clinical and pathological levels; nevertheless, no conclusive molecular markers with either prognostic or diagnostic value have been described so far. At the molecular level, the paediatric OS is a puzzle of different genetic alterations, namely point mutations or short deletions affecting 13q (RB1 gene), 17p (TP53 gene), 3q and 18q.

*Aim:* To analyse the presence of genetic alterations at 13q, 17p, 3q and 18q and to relate them with the prognosis of a group of paediatric osteosarcomas.

*Subjects and methods:* DNA was extracted from tumour tissue and peripheral blood of 37 OS patients (16 females, 21 males). Microsatellite markers screened: introns 2 and 20 of the RB1 gene (13q), intron 1 of the TP53 gene (17p), markers D3S1246 and D3S1212 (3q) and D18S42 (18q). The presence of point mutations affecting exons 5–8 of the TP53 gene was screened by DGGE analysis.

*Results:* In our series, 35% of the OS tissues carried mutations at the TP53 locus, and the analysis of microsatellite markers yielded the following results.

| | TP53 | RB1 | D3S1246 | D3S1212 | D18S42 |
|---|---|---|---|---|---|
| Non Informative | 37.% | 23.3% | 0.% | 50.% | 4.8% |
| Normal | 37.1% | 50.% | 90.5% | 45.% | 66.7% |
| Altered | 25.9% | 26.7% | 9.5% | 5.% | 28.6% |

The marker of higher prognostic value in our series is the deletion of the RB1 gene, which is associated with reduced disease-free survival ($P$=0.008). Also associated with poor prognosis and reduced survival were the histological subtype ($P$=0.0066) and the presence of metastasis at diagnosis ($P$=0.0006).

*Conclusions:* The presence of alterations at 17p, 13q, 3q and 18q is a frequent event in the carcinogenesis of paediatric OS. The homo- or hemizygous deletion of the RB1 gene, the presence of metastasis and the chondroblastic histology are associated with reduced survival in our series. Other molecular parameters screened but less frequently encountered in our series were mutation of p21WAF1, deletion and methylation of p16INK4 and RB1 promoter methylation.

**Prognostic value of P-glycoprotein in high-grade osteosarcoma: a critical evaluation**

MASSIMO SERRA[1], GEMMA REVERTER BRANCHAT[1], KATIA SCOTLANDI[1], GIUSEPPE FILIPPONE[1], MARIA CRISTINA MANARA[1], STEFANIA BENINI[1], STEFANO FERRARI[2], FRANCO BERTONI[3], CRISTINA FERRARI[1], GAETANO BACCI[2] & PIERO PICCI[1]

*([1]Laboratorio di Ricerca Oncologica, [2]Servizio di Chemioterapia and [3]Servizio di Anatomia Patologica, Istituti Ortopedici Rizzoli, Bologna, Italy)*

Multidrug resistance (MDR) associated with increased expression of P-glycoprotein has been demonstrated to play a key role in the chemotherapy unresponsiveness and clinical outcome of patients with high-grade osteosarcoma. However, a general consensus conclusion has been difficult to reach, owing to the variable results published by different research groups. Several factors may be taken into condideration to explain conflicting data, including differences in methods for sample preparation, fixation, immunostaining, analysis of immunohistochemical results, heterogeneity of clinical treatments. Aim of this study was to assess P-glycoprotein expression in different series of high-grade osteosarcoma patients in order to evaluate the influence on the immunostaining of different methods for sample preparations and to determine the prognostic value of P-glycoprotein in relation to the different drugs employed in the chemotherapeutic protocols. By using immunohistochemistry with three different, mutually exclusive monoclonal antibodies (JSB-1, MRK16, C494), P-glycoprotein expression was analyzed on 162 primary, non-metastatic, high-grade osteosarcomas, treated with chemotherapy protocols which included doxorubicin, methotrexate, cisplatin and ifosfamide. Immunohistochemical results were correlated either with clinical outcome and sample preparation. Experimental data confirmed that P-glycoprotein overexpression is one of the most important adverse prognostic factors only in osteosarcoma patients treated with chemotherapy protocols in which doxorubicin is the key drug. Concerning the methodology, this study showed that: (1) sample fixation and antigen preservation must be carefully controlled; (2) tissue-specific controls and standardized human osteosarcoma MDR cell lines are essential for calibrating the detection method and the subsequent scoring of clinical samples; (3) use of two or more anti-P-glycoprotein antibodies improve the reliability of the immunological detection. All these information must be taken into consideration before considering the possible use of P-glycoprotein as the basis for drawing innovative therapeutic regimens.

**Methylthioadenosine phosphorylase (MTAP) gene deletion affects chemotherapy drug function in osteosarcoma**

J.M. GARCÍA-CASTELLANO, C.P. JUNG, J.H. HEALEY, B. MAZZA, A. HUVOS, J.R. BERTINO, P. MEYERS & R. GORLICK

*(Memorial Sloan-Kettering Cancer Center, New York, USA)*

*Aim of the study:* Investigation of biological factors related to chemotherapy resistance might identify prognostic factors that allow stratification of patients at diagnosis into poor and good risk subgroups. The protein MTAP is a biological marker that might be exploited therapeutically. MTAP cleaves a subproduct of polyamine synthesis (MTA) into adenine and methylthioribose-1-phosphate. It is known that the absence of MTAP function: (1) makes the cells more susceptible to purine *de novo* synthesis inhibitors; and (2) increases the MTA cellular concentration, which exerts an apoptotic effect. There is no prior works showing in osteosarcoma cells the influence of MTAP status on cell behavior. We hypothesized that: (1) osteosarcoma MTAP-negative cell lines are more sensible to the action of purine *de novo* synthesis inhibitors; and (2) there will be more apoptotic response in the these MTAP-negative cells.

*Methods:* Two different osteosarcoma cell lines regarding the MTAP gene status were used: HOS, which has a homozygous deletion of this gene, and U2OS, which is wild-type for this gene. The response to a *de novo* synthesis purine inhibitor, L-alanosine, and its $IC_{50}$ was evaluated by means of a cytotoxicity assay. Cell growth inhibition was tested by XTT/PMS assay after 72 h of culture and expressed as percentage of survival. The apoptotic response of the cells was determined by staining nuclear chromatin by means DAPI. The percentage of apoptosis was determined by counting more than 7000 cells and scoring for the incidence of apoptosis using a fluorescent microscope. The statistical analysis was determined by the two-sided *t*-test.
*Results:* In the cytotoxicity assay, the $IC_{50}$ of HOS cells was between 5- and 10-fold less than in the U2OS cells. Conversely, the percentage of apoptotic cells was 5-fold greater in the HOS cells than in the U2OS cells. The difference were in all cases statistically significant ($P<0.001$).
*Conclusions:* Our results in osteosarcoma cell lines prove for first time that: (1) MTAP-deficient cells are more sensible to the action of L-alanosine than the MTAP-positive cells; (2) apoptotic response is more accentuated in the MTAP-deficient (HOS) than in the MTAP-positive (U2OS). These results show that osteosarcoma treatment and prognosis might be improved regarding to MTAP status.

### Session 2: Case presentation

### Osteogenic sarcoma in pregnant women

M. SAN JULIÁN[1], J.I. BILBAO[2], G. LOPEZ[3] & J. CA ADELL[1]
*(Departments of [1]Orthopaedic Surgery, [2]Radiology and [3]Obstetrics, University of Navarra, Pamplona, Spain)*

A 30-year-old pregnant woman consulted at our Clinic after being diagnosed with osteoblastic osteosarcoma in the distal right femur in January 1990. The pregnancy was at the fifth month, without problems. No metastases were found at the moment of diagnosis. The doctors who made the diagnosis advised her an abortion as the first step of the treatment, based both on the probabilities of influence of the pregnancy on the tumour growth and on the probabilities of foetus damage due to chemotherapy. In addition they advised an amputation as local treatment for the tumour.
*Possibilities:* (1) abortion, amputation and postoperative chemotherapy; (2) abortion, neoadjuvant chemotherapy, limb salvage and postoperative chemotherapy as usual; (3) neoadjuvant chemotherapy as usual (cisplatinum, methrotrexate and doxorubicine), abortion and limb salvage surgery, and postoperative chemotherapy; (4) neoadjuvant chemotherapy excluding methrotrexate, cesarean delivery after 9–12 weeks, limb salvage surgery and postoperative chemotherapy; (5) neoadjuvant chemotherapy as usual, limb salvage, postoperative chemotherapy and normal vaginal delivery at the end of pregnancy if possible.

### Management of a limb-length discrepancy after intercalary resection of the femur using Ilizarov device and massive bone allograft: a case report

P.A. DAOLIO, G. OLDANI & S. MAPELLI
*(Surgical Oncology Department, G. Pini Orthopaedic Institute, Milano, Italy)*

The authors present the case of an 8-year-old girl affected by osteosarcoma of the distal femur treated with polychemotherapy and intercalary resection of 15 cm. The reconstruction between the residual distal epiphysis and diaphysis was performed with an acrylic cement spacer fixed to the bone by a lateral blade-plate and a medial right plate screwed in the cement.
Margins were wide. Necrosis was >90% (stage III, Rosen). Functional result was good but with a final maximum flexion of the knee of 50°.
The implant failed after 7 years because of the breakage of the plates. The 15-year-old patient was CDF and had a 6.5-cm shortening of the limb.
The discussed solutions were: implant of a modular knee prosthesis or of a massive allograft, but with these limb lengthening would not have been possible. In order to solve the problem of shortening, the authors considered the possibility of applying the Ilizarov device, but its distal fixation should involve also the proximal tibia with a worsening of knee function.
Finally the authors chose an original solution consisting in the fixation of the Ilizarov device distally, in acrylic cement, and proximally in the femoral diaphysis after the removal of the corresponding portion of the broken plates and screws. The lengthening was perfomed between the femoral diaphysis and the cement spacer and required 2 months. Then a massive allograft of adequate length, fixed with a blade-plate, was implanted. The patient is still CDF and after 1 year fusion is achieved distally and advanced proximally, maintaining the same ROM of the knee.

### Session 3: Late effects of surgery, chemotherapy and radiotherapy in children with bone sarcomas

### Comparison of the outcome of conventional osteosarcoma at two specialist international orthopaedic oncology centres

S. FORD, A. SAITHNA, R.J. GRIMER & P. PICCI
*(The Royal Orthopaedic Hospital, Oncology Service, Birmingham, UK)*

*Objective:* To determine prognostic value of patient and treatment parameters in management of osteosarcoma, and whether these parameters are equally important across international boundaries.
*Design:* Retrospective, cross-sectional study of patients ($n$=428) diagnosed with around-knee osteosarcoma, between 1990 and 1997 at specialist orthopaedic oncology centres; Birmingham (UK) and Bologna (Italy). Disease-free survival (DFS) and overall survival (OS) assessed by Kaplan–Meier, Fisher's PLSD and Cox proportional hazard regression.
*Results:* DFS and OS were 43 and 60% at 5 years in Centre 1 and 56 and 73% at Centre 2, respectively. Median survival was 108

weeks at Centre 1 and 136 weeks at Centre 2. A significant difference in DFS and OS was demonstrated between the centres ($P$=0.0019 and $P$=0.0280, respectively). The most important prognosticators were raised alkaline phosphatase ($P$=0.002 and $P$=0.0019), degree of chemotherapy induced necrosis ($P$=0.0001 and $P$=0.0002) and tumour volume >150 cm$^3$ ($P$=0.0037 and $P$=0.0057). The most significant combination of prognosticators was alkaline phosphatase and tumour necrosis. Chemotherapy regime was found to have significantly different outcome in DFS and OS.
*Conclusions:* Prognosticators were shown to have differing value across international boundaries. Chemotherapy regime was implicated as a major factor in explaining the survival difference between centres.

**Results of treatment of osteosarcoma in children and youths according to the EORTC chemotherapy regimen in the Polish Paediatric Solid Tumours Group**

W. WOZNIAK, J. KIJOWSKI, A. SZAFRA SKI, A. CHYBICKA, J. BOGUSAWSKA-JAWORSKA, J. BOHOSIEWICZ, J. KOWALCZYK, P. KOLECKI, M. KORZON, M. LIEBHART, M. RYBAK, M. WYSOCKI, W. GOLÊBIEWSKI, B. KAZANOWSKA, M. RYCHLOWSKA, T. IZBICKI, M. KUCZABSKI, J. WÊCLAWEK-TOMPOL, K. KATSKI, W. MADZIARA, M. S. POPADIUK, BORUCZKOWSKI & M. LEDA
*(Polish Pediatric Centers for Osteosarcoma Treatment)*

*Aim and methods:* In the period 1991–2000, 98 patients with osteosarcoma were treated according to the EORTC chemotherapy regimen in the centres of the Polish Paediatric Solid Tumour Group, mostly in the National Research Institute of Mother and Child. In July 1998, a comparison trial started: its main goal is to compare in a standardized way the efficacy of two osteosarcoma chemotherapy regimens (EORTC and the adopted French HD-MTX protocol (HD-MTX+DOXO/IFO+ETO). They were both widely used in Poland but never in the same clinics, with a longer 'tradition' of the French one; therefore, we tried to find the better (or at least not worse) one in terms of efficacy, safety and economy. Until today, 28 patients were randomised to the EORTC treatment in this trial.
*Results:* As the primary site, we encountered femur (53%), tibia (28%), humerus (11%), pelvis (3%), cranium (2%), fibula (2%) and radius (1%). Fifteen percent (14 patients) were diagnosed with initial metastases. Ablative surgery was performed in 32 patients (35%), whereas the rest (60–65%) have had different forms of limb salvage or reconstructive surgery applied. Forty-one patients had measurement of percentage tumour necrosis. The range was 0–100%, median value was 60%. At this point no further conclusions have been made; however, we quite doubt if the percentage of tumour necrosis can be a useful prognostic factor.
*Conclusions:* In the whole group we encountered five relapses (one patient is alive 25 months), two toxic deaths. Generally, 63/98 patients (66%) remain alive. From 36 patients (29 of them with localized disease) with a follow-up over 5 years, 17 are alive (47%), and the patients with localized disease in this group have a survival of 55% (16 patients). This gives an EFS 0.47 with a follow-up of over 100 months.

**Hypomagnesemia secondary to chemotherapy for bone sarcomas may cause neuropathy.**

M. GABOLI, P. BASTERO, A.M. ROMERO, E. RUZA, A. PATI O, A. DIEZ, S. RAGGIO, C. MATA, M. SAN-JULIÁN & L. SIERRASESÚMAGA
*(Departments of Pediatrics and Orthopedics, University of Navarra, Pamplona, Spain)*

Secondary effects of bone tumor chemotherapy include tubular injury of the kidney and metabolic toxicity, which may result in an abnormal plasmatic level of magnesium. On the other hand, peripheric neuropathy is a very well known side effect of many antineoplastic drugs.
*Aim:* To assess whether hypomagnesemia *per se* may cause polyneuropathy in patients who underwent chemotherapy for bone sarcomas.
*Methods:* 126 clinical records of patients treated with chemotherapy for bone sarcomas (range 9–18 years) were analyzed retrospectively for chemotherapeutic treatment, plasmatic levels of magnesium and neuropathy. Neuropathy was clinically defined as persistence of at least one of the following: paresthesias and difficulty of movement in the extremities, muscle cramps or pain.
*Results:* 43 patients (34.12% of those analyzed) presented hypomagnesemia in at least one determination during chemotherapeutic treatment: 18 showed neuropathy; two had seizures. Hypomagnesemia was observed in these 20 patients while recording the neurological symptoms. Electroneurophysiology studies confirmed neurological alterations in nine cases and showed a mixed polyneuropathy of the extremities, affecting preferentially hands and feet, mainly with axonal damage. Supplements of magnesium caused a rapid relief of the symptoms and changes in the neurophysiology studies. Plasma levels of magnesium, without diet supplements, were found to be low for years after the end of chemotherapeutic treatment.
*Conclusion:* Hypomagnesemia, as a side effect of chemotherapeutic agents may cause polyneuropathy. Routine determinations of magnesium plasmatic levels during chemotherapy allow early detection of hypomagnesemia that, promptly treated, may prevent occurrence of polyneuropathy.

**Analysis of the side and late effects of treatment and clinical outcome in patients with osteosarcoma and Ewing's sarcoma**

M. GABOLI, E. RUZA, A. PATI O, P. BASTERO, A.M. ROMERO, A. DÍEZ, P.FIZ, T. BARBOSA, M. SAN-JULIÁN & L.SIERRASESÚMAGA
*(Departments of Pediatrics and Orthopaedics, University of Navarra, Pamplona, Spain)*

Improvement of survival of pediatric patients with bone tumors has changed the course of these diseases and the pattern and type of late effects of treatment.
*Aim:* To achieve a better knowledge of the clinical evolution of children with bone tumors in order to improve their quality of life.
*Methods:* Clinical records of 70 children with osteosarcoma (OS) (32 females and 38 males) and 44 children with Ewing's sarcoma (ES) (18 females and 26 males) were analyzed.
*Results:* Mean age at diagnosis was OS 13.9 years (4.01) and ES 13.3 years (4.3). Main primary tumor localizations in OS were 41.7% femur and 40.3% tibia; in ES were 13% femur, 20.4% tibia and 18.5% extraskeletal. Median duration of treatment was 12 months (11–15) and 11 months (10.5–15.5) in children with OS and ES, respectively; median time of hospitalization was 6.7 months (4.93–9.9) in OS patients and 5 months (3.4–7.6) in ES patients. Mean disease-free survival time was 4.8 years (3.9) and 3.8 years (3.6) in OS and ES patients, respectively. The following table shows the most frequent side effects and complications:
*Conclusion:* The knowledge of the clinical history of bone tumors in children allows the early detection of side effects of treatment and a better quality of life, during therapy and while in remission.

| % | OS | ES | % | OS | ES | % | OS | ES |
|---|---|---|---|---|---|---|---|---|
| Myelotoxicity | 95.6 | 90.9 | Renal diseases | 22.1 | 7.3 | Sepsis | 11.8 | 9.8 |
| Infection | 33.8 | 34.2 | Osteomielitis | 19.1 | 12.2 | Psychiatric | 8.8 | 9.8 |
| Heart disease | 27.9 | 31.7 | Amputation | 13.2 | 14.6 | Hypoacusia | 13.2 | 2.4 |
| Neuropathy | 23.5 | 19.5 | Liver disease | 17.6 | 9.8 | Relapse | 17.6 | 4.8 |
| Months at diagnosis | 21.4 | 16.7 | Months in treatment | 7.1 | 9.5 | Months in remision | 22 | 19.5 |
| Lung months | 68.2 | 46.3 | Bone months | 17.1 | 27.8 | Death | 17.2 | 20.4 |

**Decision making in orthopaedic oncology**

R.J. GRIMER & P. COOL
*(Royal Orthopaedic Hospital Oncology Service, Birmingham, UK)*

*Aim:* To provide a quantitative system for evaluation of the best surgical intervention for patients with bone tumours.
*Method:* Deciding what is best for an individual patient with a bone tumour is becoming more difficult as the choices become greater. One has to weigh the advantages of different types of surgical reconstruction with the time it takes to recover from the various procedures and the risk of complications attached to each type of surgical procedure both in the short and long term. Also, one has to consider whether there is an increased risk to a patient survival by performing a limb salvage procedure to try and preserve function as opposed to doing an amputation. In order to try and quantify the risks and benefits of various types of procedure we have developed the concept of FUNLYs (FUNctional Life Years) which is loosely based on the well established Quality Added Life Year (QALY) used by health economists. The FUNLY uses the MSTS score now available for many procedures and the published risks of complications and further surgery for various procedures to calculate relative values for different procedures.
*Results:* FUNLY scores can be worked out for different tumour types and different treatment methods. A computerised web-based program will be demonstrated highlighting its use both in situations where there are multiple options for treatment and also in a situation where there are large differences in function but significantly greater risks of failure. For an osteosarcoma of the distal femur results indicate: endoprosthetic replacement 5.38 FUNLYs, allograft 5.21, rotationplasty 5.6, arthrodesis 3.6, distraction osteogenesis 5.2, amputation 4.0.
*Conclusion:* FUNLYs offer a logical method of comparing different interventions to highlight benefits and deficiencies of each.

**Distal femur intercalary resection and reconstruction in high grade bone sarcoma**

D. DONATI, S. GIACOMINI, E. GOZZI, M. MANFRINI & M. MERCURI
*(Oncologic Department, Instituto Ortopedico Rizzoli, Bologna, Italy)*

The aim of the study was to define how much closer and safer we can cut the osteotomy line in the distal aspect of the femur in intercalary resections. We reviewed all the cases of high-grade bone sarcoma located in the femoral diaphysis, selecting only those with a distal cut within 8 cm from the knee joint. There were 58 patients divided in three groups: Group A (mean age 22 years), 20 cases performed from 1978 to 1987 and reconstructed with temporary cementation or autografts. Group B (mean age 17 years), 25 cases performed from 1985 to 1998 reconstructed with allograft alone and Group C (mean age 12 years), 13 cases, same period, reconstructed with allograft and vascularized fibula. In Group A the osteotomy line was at a mean distance of 5.6 cm, and only in three of them was the growth plate considered active at time of surgery. The margins were judged inadequate in three cases, therefore two recurred. Eight cases survived long enough for evaluation; however, four of them had a failure of the reconstruction and only four are still working with satisfactory result (mean follow-up 182 months). In Group C, 5 cm mean distance from the joint, up to nine cases (70%) were still skeletally immature at time of surgery. Inadequate margins were noted in two cases, and one of these and one other recurred (15%). Complications were infection in one case, fracture in four cases (30%), and non-union in three cases: three reconstructions failed (23%) while the other scored satisfactory eight cases, and fair in two cases (mean follow-up 70 months). In conclusion, saving the articular joint in distal femur resections brings a high number of satisfactory results at follow-up. Complications and failure (including recurrence) occurred in a fairly high number of cases. The choice of the vascularized fibula allows a closer cut to the growth plate; however, complications, such as local recurrence and fracture, can be expected.

**Late results in child knee reconstruction for bone sarcomas**

M. MANFRINI, L. CAMPANACCI, M. DE PAOLIS, E. GOZZI, E. GUERRA & M. MERCURI
*(Istituto Ortopedico Rizzoli, Bologna, Italy)*

From 1984 to 1999, 214 children with a sarcoma of the distal femur or proximal tibia (195 osteosarcoma, 15 Ewing's sarcoma and four MFH) were surgically managed at Istituto Rizzoli. A limb salvage was performed in 153 cases (72 %), A1 rotationplasty in 33 cases (15%) while amputation was chosen in 28 cases (13%). Late results were studied in 86 patients, alive more than 3 years after the reconstruction (mean age 11; range 7–13; mean follow-up 98 months). There were 32 arthrodeses, 20 prostheses, two allograft/prosthesis composites, 14 osteoarticular allografts, while 18 cases had intercalary juxta-articular reconstructions. In the arthrodeses, five patients had a secondary amputation between 5 and 144 months after the reconstruction because of early or late infection. Three other cases were converted to prostheses after mechanical failure. In other eight patients, at least one lengthening procedure was performed. In the remaining cases the mean final shortening was almost 8 cm in males and 4 cm in females. In prostheses, there were one total femur lengthening, one standard total femur, 15 distal femur and three proximal tibia uncemented prostheses. In five cases a smooth stem was used to minimize the damage to tibial physis. Two patients were amputated because of late infections. Three patients had stem fracture and were converted to total femur prosthesis. Four patients had lengthening procedures on the prosthesis. The remaining knees had a mean shortening of 5 cm in males and 3 cm in females. Osteoarticular allografts were implanted both in distal femur or proximal tibia with different results. All six proximal tibias failed and were converted in composite prostheses. One late infection ended with the amputation. Two femur reconstructions were converted to prostheses. In the six patients with femur allografts still in place, final shortening was 4.5 cm in males and 2 cm in females. In intercalary juxta-articular reconstructions (mean follow-up 90 months), two patients presented postoperative deep infections: one had the implant removed and the bone reconstructed by Ilizarov technique, the other one ended in above-knee amputation. In the 16 patients with the original implant, the mean shortening was 2 cm, both in males and females. Functional satisfactory results (Excellent or Good according to the Enneking System) increased from 25% in arthrodeses to 55% in prostheses, 57% in osteoarticular grafts and 67% in intercalary reconstructions.

**The risk of amputation following limb salvage surgery with endoprosthetic replacements**

L. JEYS, R.J. GRIMER, S. CARTER & R.M. TILLMAN
*(Royal Orthopaedic Hospital Oncology Service, Birmingham, UK)*

*Introduction:* Endoprosthetic replacements (EPRs) are one of the most commonly used types of limb salvage following surgical excision of bone tumours. The advantage of EPRs are their initial reliability and the rapid restoration of function along with their ready availability. The problems with eprs are the long-term problems of wear, loosening, infection and mechanical failure. This paper assesses the risk of amputation following EPR.
*Patient and methods:* We retrieved data on all patients who had an amputation after a previous EPR and looked at the reasons for this. By comparing this group with patients who had not undergone amputation we estimated the risks of amputation and stratified this by type of prosthesis, diagnosis and complication.
*Results:* A total of 1262 patients have undergone epr surgery at our centre in the past 34 years: a total of 6507 patient years of follow-up; 112 patients have had subsequent amputation (8.9%). The reasons for amputation were local recurrence in 71(64.4%), infection in 38 (33.9%), mechanical failure in two (1.8%) and continued pain in one case (0.8%). The risk of amputation was greatest in the proximal tibia 15.5% ($n$=38/246), followed by pelvis 10.2% (5/49), and femur 7.4% ($n$=58/784), whilst the risk of amputation was least in the humerus at 6.4% ($n$=11/182). The risk of amputation was highest in the patients diagnosed with rarer tumours (33% [$n$=4/12]), mfh (17.7% [$n$=9/51]) and soft tissue sarcoma (14% [$n$=10/71]). The time to amputation varied from 2 days to 16.37 years, with a mean of 31 months. The median time to amputation from EPR was 32 months for infection and 13 months for local recurrence. The risk of amputation decreased with time, although 10% of the amputations took place more than 5 years after implantation.
*Conclusion:* The greatest risk of amputation is in the first 5 years and is due to local recurrence, whilst infection poses the next greatest threat. The risk decreases with time. Attempts to control both local recurrence and infection will decrease the need for amputation. Late failure of the EPRs, even in young patients does not seen to be a major cause of amputation thus far.

**Long-term results of expanding protheses for limb salvage surgery of children**

G. DELEPINE[1], F. DELEPINE[2] & N. DELEPINE[1]

*([1]Oncologic Ped. Sce Hop., Bobigny, France, [2]Rouen, France)*

*Introduction:* Conservative surgery for young children with bone sarcoma of lower limb remains a challenge. In 1985 we proposed an expandable prosthesis and present here our long-term results.
*Patients:* 44 patients (20 males and 24 females aged 4–28 years) with tumors of the limbs were treated by our team between 1984 and 1999. Histology was mostly osteosarcoma (32) and Ewing's sarcoma (9). Locations were distal femur in 30, upper tibia in five, total femur in five and proximal femur in four. Thirty were first hand patients (28 with localized disease and two already metastatic) *en bloc* resection. The remaining 14 patients were referred to us after induction therapy, with progressive disease, metastase (3) or local recurrence (1).
*Method:* In 14 patients the expanding prosthesis was inserted immediately after the resection, in eight during the following year and for the other 22 patients later to treat a length discrepancy. One hundred and seven sequences of lengthening have been performed in 40 patients. All patients were followed-up by their surgeon and their chemotherapist every 3 months during 2 years, then every 6 months for a further 2 years and yearly thereafter.
*Results:* 6 patients died from illness. All others are disease-free survivors with a median follow-up of 91 months(maximal 192; minimal 6). Half (22) of the patients are adults. The average lengthening is 4.07 cm (minimal 0.5; maximal 12). Half of the patients had to be re-operated for complications. Deep infection occurred in 10 patients (22%) resulting in amputation for three. According to EMSOS criteria, the functional result is excellent in 14, good in 15, fair in 10 and poor in five.
*Conclusion:* Long-term results of lengthening prostheses confirm that this procedure is an excellent alternative to amputation and permits a functional limb in nearly 90% of patients. The most severe complication is deep infection, underlining the interest of previous generations on growth with minimally invasive lengthening.

**Infection after extensible endoprosthetic replacements for bone tumours**

M.V. BELTHUR, R.J. GRIMER, S.R. CARTER & R.M. TILLMAN
*(The Royal Orthopaedic Hospital, Birmingham, UK)*

*Aim:* To analyze the risk factors, causes, bacteriology of these infections and to review our experience with the treatment of 19 patients with infected prostheses.
*Materials and methods:* 123 patients were treated with extensible endoprostheses following resection of primary bone tumours of the distal femur, proximal tibia, proximal femur and humerus between 1983 and 1998. Three types of prostheses, which differed in the lengthening mechanism used, were implanted. Nineteen of these were diagnosed to have deep infection. Details of the patients, clinical features and investigations were secured using the bone tumour unit database and using patient records. Bacteriology, risk factors and causes were analyzed. Patients were divided into three groups: group 1, five patients were treated with a single stage revision; group 2, 13 patients were treated with a two-stage revision procedure; group 3, one patient was treated with a primary amputation.
*Results:* The overall incidence of infection was 15.45%. The incidence of infection at the proximal tibia and distal femur was 28 and 14.3%, respectively. The incidence for the three types of prostheses was 20.85% for the ball bearing (type 3), 18.75% for the C-collar (type 4) and 16% for the worm wheel (type 5). The average infection delay was 40.6 months. *Staphylococcus epidermidis* was the most common organism isolated. The most common clinical features were pain and swelling around the prostheses, with discharging sinuses being more common around the proximal tibia. Infection in almost all cases was immediately preceded by an operative procedure or by a focus of infection elsewhere in the body. Group 1 patients: 20% success rate; Group 2, 84.6% success rate. Amputation was the salvage procedure of choice for failed revision procedures and was necessary in four out of five patients in Group 1 and two out of 13 patients in Group 2. The mean MSTS functional score was 83.3% (range 63–97%) in patients in whom the infection was controlled.
*Conclusion:* The incidence of infection is high following extensible endoprostheses, especially the proximal tibia. The type of prosthesis used and the number of lengthening procedures constitute significant risk factors. Two-stage revision is successful in controlling infection in a majority of these cases.

**Deep infection of tumor endoprostheses**

G. GOSHEGER, D. BÜCHTER, A. HILLMANN & W. WINKELMANN
*(Department of Orthopaedics, Westfälische Wilhelms-Universität, Münster, Germany)*

In a retrospective study of 230 patients having undergone implantation of a tumorendoprosthesis, we tried to figure out reasons for deep infection and looked for strategies to minimize deep infection of tumor endoprostheses: 145 MUTARS® endoprostheses and 85 KMFTR® endoprostheses were implanted between March 1991 and December 1998. The results of the present study show that the use of gentamycin collagen floss (Sulmycin® implant) could significantly reduce infection ($P$=0.03, $\chi^2$-test). The additional application of systemic antibiotics (cephalosporines, third generation, e.g. Rocephin® or Fortum®) could further reduce the risk of infection. Mandocef® (cephalosporines second generation) was significantly less efficient. The postoperative antibiotic regimen has to be sensitive especially against staphylococcus species (69.7% of germs). Further factors which significantly influence the rate of infection were the amount of blood loss, the operation time and the diagnosis. Laminar air flow, the amount of preoperative leucocytes and cementation of the endoprostheses had no significant influence on the rate of infection ($P$>0.05).

**Staged revision for infected reconstruction after limb salvage surgery for bone tumors**

N. FABBRI, D. DONATI, S. GIACOMINI, M. MANFRINI & M. MERCURI
*(Department of Musculoskeletal Oncology, Istituto Rizzoli, Bologna, Italy)*

*Study aim:* The purpose of this study was to evaluate the results of a staged revision technique in the treatment of deep infection after limb salvage surgery for bone tumors and to identify factors possibly affecting the outcome.
*Materials and methods:* A retrospective study of 19 consecutive patients with an infected bone tumor reconstruction treated at our Institution in the period 1986–1997 was undertaken. All the patients underwent staged revision (two stages in 13 cases, three stages in 5, four stages in 1) using one or more antibiotic-loaded cement spacers after debridement and partial (10 cases) or complete (nine cases) removal of the original implant. Postoperatively, all the patients received oral or parenteral antibiotics for a minimum of 4 weeks. Delayed reimplantation was performed in 15 cases, average time to reimplantation being 7 months (4–14). A minimum follow up of 3 years was available in all patients. Cultures identified *S. epidermidis* in 12 cases (63%), *S. aureus* in four (21%), mixed organisms in two (11%), and were negative in one case (5%), despite clinical evidence of infection.
*Results:* At a minimum follow-up of 3 years, 13 patients were continuously infection-free (68%) while six relapsed (32%). Two of the six relapses were cleared by amputation while four remained infected. Average functional result of infection-free patients according to the International Society Of Limb Salvage (ISOLS) was 71% (21.2 points). Success rate was 89% (eight out of nine patients) when a complete implant removal was accomplished and only 50% (five out of 10 patients) with partial removal. Success rate was 82% against methicillin-sensitive organisms, 60% with methicillin-resistant and 0% when mixed organisms were present. When a complete implant removal was combined with *Staph. epidermidis* a 100% success rate was obtained. Success rate was 73% using vancomycin-impregnated cement spacers and 50% with other antibiotics. Treatment was successful in 100% cases (six out of six patients) when negative cultures were obtained at the time of reimplantation and in 50% cases (three out of six patients) when cultures were positive.
*Conclusions:* Staged revision with antibiotic-loaded cement spacer for infected bone tumor reconstruction is a demanding and expensive technique requiring prolonged inability. Overall success rate in this series approaches 70%. Complete removal of the infected implant, microbiology, appropriate antibiotic selection, and negative cultures before reimplantation are crucial.

**Our experience on postoperative radiationtherapy for low-grade soft tissue sarcomas**

R. CAPANNA, G. BELTRAMI, P. CALDORA, M. MELA°, P. OLMI,° M. PERTICI°, R. DE FELICE & R. PASSALACQUA
*(Chirurgia Ortopedica Ricostruttiva CTO, and °U.O. Radioterapia, Florence, Italy)*

Although wide excision alone is generally considered sufficient for the local control of low-grade soft tissue sarcomas, adjuvant radiation therapy being recommended only for inadequate surgical margins, recurrences may be observed following both wide and marginal surgery. We present our experience on 77 cases of low-grade soft tissue sarcomas, comparing three series of patients, treated by different approaches: Group A (13 cases) surgery alone; Group B (16 cases) surgery plus external radiation therapy; Group C (48 cases) surgical excision, brachytherapy, and external beam radiation therapy (45 cases) or surgery plus brachytherapy alone (three cases). The groups were comparable for tumor size and surgical margins. At an average 48-month follow-up, metastases occurred in seven cases (9%) of the overall groups. Local recurrences occurred in four patients of Group A (31%), no patients of Group B and two patients of Group C (4%). The recurrences of Group A were resected in three cases and amputated in one, while that in Group C were resected in one and untreated in the other (severe metastatic disease). In Group A, only one case of major complications (one wound slought, healed after surgical reprises) was observed. In the radiotherapy groups (B+C), nine major complications (14%) were found: three fractures, three wound slought, three nerve palsy. All these complications were observed in patients in Group C. A successful conservative treatment was done in seven of these patients, while two patients had to be amputated. In our experience, radiotherapy showed to be efficient in improving the local control of low-grade soft tissue sarcomas (31% of recurrence in Group A vs. 3% in Group B+C). No significant differences on local control were observed between Group B and C. Limb salvage surgery had a failure rate of 8% (1/13) in the surgery alone group, while it was 3% (2/64) in the groups of patients treated by surgery plus radiotherapy. Our actual trend is to use conventional postoperative radiotherapy.

**Analysis on 141 consecutive soft tissue sarcomas, treated by surgery plus brachytherapy**

R. CAPANNA, G. BELTRAMI, D.A. CAMPANACCI, P. CALDORA, L. CIAMPALINI, M. MELA°, P. OLMI° & M. PERTICI°
*(Department of Orthopaedic Oncology, °Department of Radiotherapy, Florence, Italy)*

Although surgical excision plus brachytherapy and/or adjuvant radiotherapy is generally highly suggested to achieve local control in soft tissue sarcomas, complications related to radiotherapy may be more and more often observed. We report our experience on 141 patients, treated in our Center from July 1990 to November 2000. High-grade sarcomas were represented in 93 patients, low-grade in 48. The upper limb was involved in 38 cases, lower limb in 96, trunk in seven. All patients were treated by limb-sparing surgery and had brachytherapy (average 35 Gy). Postoperative external radiotherapy was associated in 128 cases, while preoperative radiotherapy was given in five cases (average 45 Gy); brachytherapy alone was employed in eight patients. Adjuvant chemotherapy (epirubicin plus ifosfamide) was associated in 31 patients. At an average follow-up of 54 months (from 4 to 120), 95 patients (67%) continued to be disease free, 11 (8%) had no evidence of disease (after treatment of recurrence or distant metastasis), 13 (9%) were alive with metastatic disease, 17 (12%) were dead of disease, five (4%) were dead from other causes. Local recurrence appeared in seven patients (5%): five were treated by conservative surgery and two were amputated. Some severe complications related to radiotherapy were observed in 14 patients (10%): fractures (5), wound slough (6), major nerve palsy (3). These were treated conservatively in all but three cases (amputated). Regarding the overall clinical results (MSTS), 110 patients (78%) were rated as satisfactory, with 31 (22%) as unsatisfactory (18 fair, 13 poor). Despite some adverse local impact, brachytherapy and external radiotherapy showed to be efficacious as adjuvant in achieving a good local control of soft tissue sarcoma.

**Hemangioendothelioma of the mobile spine**

S. BORIANI, A. GASBARRINI, S. BANDIERA, E. BERTOLDI, L. MIRABILE, G. BARBANTI & F. BERTONI*
*(Department of Orthopedics and Traumatology, Ospedale Maggiore, and *Istituti Ortopedici Rizzoli, Bologna, Italy)*

*Aim:* Hemangioendothelioma (HE) of bone is a rare tumor (about 1% of primary malignant muscolo-skeletal tumors) originating from angioblastic endothelial cells. A progression of malignancy can be observed in the same tumor and in different entities from low-grade tumors, to differentiate from hemangioma, to high-grade malignancies, to differentiate from true angiosarcomas. Ten percent of HE arise from the spine, provoking vertebral collapse and/or neurological signs at the onset. Early diagnosis and staging are critical for spine function and *quod vitam* prognosis. The purpose of this study is to describe the different presentations and evolution of these entities as well as the different treatment options.
*Methods:* Ten cases of HE of the mobile spine were retrospectively reviewed: the series included four males and six females aged 21 to 64 years (average 30 years). Four cases arose from the thoracic spine, five from the lumbar, one from the thoraco-lumbar junction.
*Results:* At the final clinical examination (follow-up 6–120 months, average 46 months), six patients were continuously disease free, two were NED (35–41 months after biopsy), two died (17–84 months after biopsy). *En bloc* resection was performed in two cases and allowed for complete local and systemic control of the disease (follow-up 6–65). Intralesional excision was effective if associated with radiation in five of seven cases. Multicentric onset, a characteristic feature of hemangioendotheliomas, was observed in one case.
*Conclusions: En bloc* resection is the treatment of choice for HE, it is associated with the lowest recurrence rate, but dissemination of the disease remains at risk.

**Primary spindle cell sarcoma of bone: an assessment of outcome**

R. GRIMER, C. DOCKER & D. SPOONER
*(The Royal Orthopaedic Hospital, Birmingham, UK)*

*Aim:* To assess whether spindle cell sarcomas of bone behave like other primary bone sarcomas.
*Method and material:* 193 patients with spindle cell sarcomas of bone have been treated over the past 25 years. Diagnoses have included spindle cell sarcoma, MFH, leiomyosarcoma, fibrosarcoma and angiosarcoma. The mean age was 47 years and 121 were over the age of 40. All but three were high grade and 26 were known to have metastases at diagnosis. Ten arose secondary to irradiation or Paget's disease. The most common site involved was the distal femur followed by the tibia, pelvis and humerus. Treatment was with chemotherapy and surgery if suitable. Twenty-two percent required an amputation. Outcome in terms of local control and overall survival has been assessed to identify presenting and treating factors of significance.
*Results:* Overall survival was 52% at 5 years. Patients with metastatic disease at diagnosis, with pelvic primaries and with radiation induced sarcomas all had worse survival and local control. If these patients are excluded then survival was 60% at 5 years. Other factors of prognostic significance were age (patients <40 did better than those >40) and chemotherapy response. Local control was better after amputation than limb salvage surgery but overall survival was no different. The histological diagnosis made no difference on outcome
*Conclusion:* Spindle cell sarcomas of bone are primary sarcomas which do not fit into the categories of osteosarcoma, chondrosarcoma or Ewing's sarcoma. They tend to arise in an older age group When treated by similar principles of chemotherapy and surgery the outcome is similar to that of osteosarcoma.

**What is the price of surgery in patients with a bone tumour located in the pelvis?**

C.H. HOFMANN[1], A. HILLMANN[1], C. GEBERT, G. GOSHEGER[1], H. JÜRGENS[2] & W. WINKELMANN[1]
*(Departments of [1]Orthopaedics and [2]Hematology and Oncology. University of Münster, Germany)*

*Objective and methods:* Surgical removal of pelvic tumours with or without irradiation offers the best chance for long-term relapse-free survival. Whereas aggressive surgery will improve local control, it also needs extensive rehabilitation and the functional outcome may not be satisfactory. The aim of an prospective study was to analyse the late functional outcome and the quality of life assessment of patients with a musculo-skeletal tumour located in the pelvis. The functional evaluation score of Enneking and quality of life (EORTC-QLQ C30) were determined in a cohort of 81 patients with a pelvic tumour.
*Results:* The median follow-up time was 48 months. The surgical procedure includes hip transpositioning (HT), tumour resection and endoprosthetic replacement (ER), and tumour resection and reconstruction with allo or autograft (AR). The median Enneking score of the whole group was 18/30 (range 2–30). In patients with ER the median Enneking score was 9/30 (range 6–18) in comparison to 19/30 (range 6–27) in patients with HT and 18/30 (range 6–30) in patients with AR. In the quality of life analysis, HT had significant better results in social functioning mean 62.5 compared to 30.3 in ER and 53.1 in AR. Asking for limitations in hobbies work or their daily activities (RF2) HT faired better, mean 55.5 compared to 22.7 in ER an 46 in AR. Also in the global health status (QL2) HT faired better mean 75 (ER 51.5, AR 69). In physical functioning, HT (mean 70) and AR (mean 74) had better results than ER (mean 54.5).
*Conclusion:* The hip transpositioning states a convincing surgical procedure in patients with pelvic tumours. Better functional results in social functioning, role functioning and global health status showed that there is no psychosocial outcome disadvantage. A resection and biological reconstruction in pelvic tumours improve local control and the function and quality of life outcome will be satisfactory.

**Sciatic nerve resection: is that truly an indication for amputation?**

ISAAC MELLER, YEHUDA KOLLENDER & JACOB BICKELS
*(Tel Aviv Sourasky Medical Center, Tel Aviv, Israel)*

*Background: En bloc* resection of the sciatic nerve with a malignant tumor of the pelvis or thigh was long considered an indication for an amputation because of the anticipated poor function. The authors describe the functional outcome of a group of patients who underwent a limb-sparing surgery in spite of the need to sacrifice the sciatic nerve.
*Materials and methods:* Between 1991 and 1999, the authors treated 12 patients who underwent limb-sparing resections of a malignant tumor of the thigh, buttock, or pelvis, all of which necessitated *en bloc* resection of a segment of the sciatic nerve with the tumor mass. There were eight females and four males, ranging in age from 2 to 73 years (mean, 54 years).
*Diagnoses:* Soft-tissue sarcomas, eight; metastatic bone disease, three; primary bone sarcomas, one.
*Anatomic locations:* Thigh, seven; pelvis, four; buttock, one. Follow-up ranged from 10 to 102 months (mean, 30 months).
*Results:* At the most recent follow-up evaluation, 11 patients were ambulatory and only one patient was wheelchair bound. Of the ambulatory patients, only five patients required a walking aid (crutches or a cane); a long-leg brace was not required by any of these patients. Although all patients had an anesthetic ipsilateral foot, none had a pressure sore or required a secondary amputation. All ambulatory patients were satisfied with the functional outcome.
*Conclusion:* Provided the femoral nerve is intact, the mere necessity to resect the sciatic nerve with a given tumor of the pelvis or thigh is not an indication for an amputation.

**Inlay allograft reconstruction after resection of low-grade malignant bone tumours**

A.H.M. TAMINIAU, R.L.M. DEIJKERS & R.M. BLOEM
*(Leiden University Medical Centre. Leiden, The Netherlands)*

Advances in MRI and histopathology made hemi-cortical tumor resection with wide margins possible.
*Methods:* We performed 22 hemi-cortical resections of low-grade parosteal osteosarcoma (six), peripheral chondrosarcoma (six) and adamantinoma (10) (proximal femur (two), distal femur (six), proximal tibia (10), proximal humerus (two). Follow-up was 64 months (range 27–135), mean age 25 years (range 16–60). Focal intramedullary involvement was no exclusion criteria. The decision to perform a hemi-cortical resection depended on the possibility to obtaining wide surgical intramedullary margins based on MRI. Mean resection length was 10.9 cm (range 6–26). Resection of the circumferential cortex varied from 50 to 80%. Intra-cortical resection of part of the opposing cortex was performed. twice. Fresh deep-frozen inlay allograft (Netherlands Bone Bank Foundation), precisely cut to the defect, fixed with lag screws or plate were used on impacted allogenic/autogenic cancellous bone. Follow-up included radiological evaluation of primary site and chest. The allograft–host union was evaluated (ISOLS). Functional results were assessed at the latest follow-up using the MSTS system.
*Results:* Histology showed wide resection margins in 12 and marginal in 10. Marginal resections toward soft tissue occurred to preserve neurovascular structures (no adjuvant therapy). Marginal resection towards bone occurred twice (local adjuvant: phenol/alcohol). No signs of recurrence or metastasis were present at the latest follow-up. All allografts showed complete incorporation between 6 and 30 months (mean 15). No allograft fracture, wound complication or infection occurred. Fractures of host bone occurred 6 times (during 3 times and shortly after surgery 3 times), mainly at a sharp resection corner, managed easily. The functional results were good or excellent in all but one. The poor result was due to a stiff knee after prolonged immobilisation (inter-condylar fracture).
*Conclusion:* Hemi-cortical resection of selected cases of low-grade surface tumours, followed by inlay allograft reconstruction is a safe and highly effective procedure, not restricted by intramedullary involvement and satellite lesions. Preoperative planning with MRI is essential in assessing these wide resection margin. Although the surgical technique is demanding, the superior clinical results makes it clearly worthwhile.

**Prognostic factors in parosteal osteosarcoma**

A.H.M. TAMINIAU, P.D.S. DIJKSTRA, H.M. KROON & P.C.W. HOGENDOORN
*(Departments of Orthopedic Surgery, Radiology and Pathology, Leiden University Medical Centre, Leiden, The Netherlands)*

Parosteal osteogenic sarcoma ( juxtacortical osteosarcoma) is a low-grade malignant surface bone tumor that arises from the surface of the metaphysis of long bones. A retrospective review was conducted of 17 patients surgically treated between 1980 and 1998. The clinicopathological features especially in relation to the preoperative MRI and CT findings were analysed. Surgical staging for stage IA, IB, IIB resulted in nine, five and three patients, respectively. Local recurrence developed in three patients (range 1–8 years postoperatively) and in one patient with long metastases. Local recurrence occurred; in one patient after an open biopsy in a stage IB tumor with medulla invasion and with a positive surgical margin after resection, and two patients with a high-grade lesion of whom one had an open biopsy. A wide, marginal and positive surgical margin were found in nine, four and four patients, respectively. A dedifferentiated component was only in identified 1 patient and was not diagnosed by MRI or CT. On MRI and CT, the median ratio of circumferential cortical involvement by the tumor was one-third. Cross-sectional imaging demonstrated medullary involvement in five patients and compared to the histological findings a false-negative result in two patients. These findings, together with the estimated volume of the tumor on CT and macroscopic measurements, were not correlated to recurrence or medulla invasion. In contrast, local recurrence presented in three out of eight patients with contact between the tumor and the local neurovascular structures by MRI analysis. In conclusion, medullary invasion and amount of cortical involvement were not poor prognostic factors. However, a poor prognostic factor was a stage IIB lesion. If the radiological diagnosis, nowadays based on dynamic MRI, is a low-grade parosteal lesion a biopsy should not be performed routinely.

### Session 4: Neoadjuvant chemotherapy in soft tissue sarcomas

**Preoperative treatment of limb soft tissue sarcoma by isolated limb perfusion (ILP) with TNF/melphalan versus systemic doxorubicin-based chemotherapy: a non-randomized comparison**

I. MELLER, Y. KOLLENDER, M. INBAR, J. ISSAKOV, G. FLUSSER & O. MERIMSKY
*(Tel-Aviv Sourasky Medical Center, Tel-Aviv, Israel)*

*Background:* Chemotherapeutic cytoreduction of soft tissue sarcomas (STS) may permit less radical operations. In cases of large or multi-compartmental deep masses, or involvement of a neurovascular bundle, its downsizing is required before limb-sparing (LSS) surgery can be considered.
*Patients:* Thirty-six patients were suitable for either technique. All were treatment (chemotherapy, radiotherapy, and surgery)-naive patients. They had big and deeply seated AJCC stage IIIB limb STS, with no evidence of systemic spread or local bone involvement. All were candidates for amputation or extremely mutilating surgery. All had preserved function of heart, lung, and kidney, absence of vascular disease in the affected limb, and signed an informed consent.
*Methods:* Retrospective, non-randomized study of results achieved by isolated limb perfusion (ILP) with tumor necrosis factor- (TNFα) and melphalan versus systemic doxorubicin-based chemotherapy. In the ILP group one ILP session was given preoperatively, and surgery was performed after 6–8 weeks. In the chemotherapy group three courses of chemotherapy were administered before surgery, which was performed 4 weeks later. Adjuvant chemotherapy was given according to the histological results of the preoperative treatment.
*Results:* For ILP group (23 patients): clinical RR 85%; LSS rate 100%; free margin rate 11/23; secondary amputation rate 2/23; median percent necrosis 70; median DFS 25 months. For chemo-group (13 patients): clinical RR 40%; LSS rate 100%; free margin rate 10/13; secondary amputation rate 0/13; median percent necrosis 90; median DFS not reached yet. Differences in parameters were statistically significant only for response rate.
*Conclusions:* Neoadjuvant doxorubicin-based systemic chemotherapy. for AJCC IIIB STS achieved at least a similar necrosis rate as preoperative ILP with TNF/melphalan. The higher response rate of STS to ILP did not lead to an improved necrosis rate nor to an improved DFS.
Preoperative ILP/TNF versus systemic ADR-based chemotherapy

| Arm | Clinical RR (%) | % LSS | Free margins | Secondary amp. rate | Median % necrosis |
|---|---|---|---|---|---|
| ILP | 85 | 100 | 11/23 | 2/23 | 70 |
| ChT | 40 | 100 | 10/13 | 0/13 | 90 |
| P value | <0.001 | NS | NS | NS | NS |

**Feasibility of pre- or post-operative combined chemotherapy (CT) and radiation-therapy (RT) in adult soft tissue sarcomas of extremities or girdles (STS): a pilot study**

F. GHERLINZONI*, A. DE PAOLI#, A. BUONADONNA#, S. DEMITRI*, G. BERTOLA#, G. BOZ#, I. INNOCENTE#, M. BERRETTA# & C. ROSSI#
*(*Orthopaedics Department, Gorizia Gen. Hosp., Gorizia; #C.R.O., Aviano, Italy)*

In the authors' experience, adjuvant CT (inside a randomized trial) induces a substantial benefit in terms of DFS and OS in the treatment of STS (JCO 2001, in press). In that study, CT was planned after any local treatment and recovery; the median time from diagnostic biopsy to CT was 92 days (range 17–288). Therefore, a multimodality approach with concomitant RT&CT, whether totally pre-op or post-op, was designed to define its feasibility & toxicity.
*Methods:* Eligibility criteria were high grade (Grade 3–4 Broder), sub-fascial spindle cell and polymorphous STS, size >5 cm or any dimension if relapse; age >16 to <65 years; PS <2 ECOG; no previous CT/RT. Treatment consisted of CT and RT concomitantly administered. CT: epirubicin 60 mg/m$^2$ i.v., days 1–2; ifosfamide and mesna 3 g/m$^2$ i.v., days 1–2–3 and G-CSF 300 μg/die s.c. from day +9 to day +16; cycles were given every 21 days. RT in pre-op setting: 22 Gy/11Fr between cycles 1 and 2 and between cycles 2 and 3 of CT and a post-op boost of 16 Gy/8Fr, between cycles 4 and 5 in case of positive margins. In post-op setting: all three blocks of treatment were given.
*Results:* Seventeen patients have been enrolled, 10 as pre-op, seven as post-op. Toxicity on 41 evaluable cycles of CT was as follows: leucopenia G 3–4, 51%; thrombocytopenia G 3–4, 14%; anemia G 3–4, 9%; non-hematological toxicity G 3–4, 32% (stomatitis 4%, erithema 21%, proctitis 7%). All 10 patients treated in the pre-op setting (three still on-going) underwent conservative surgery; one patient had major complication (infected seroma). No complications were noted in the post-op group.
*Feasibility:* The average median dose intensity of CT was 84% (60–100%). RT was given at the foreseen doses and treatment time in 13/14 evaluable patients (compliance 93%).
*Preliminary conclusions:* In the pre-op setting, the time between diagnostic biopsy and start of CT was 9 days (5–34). This integrated combined modality approach allowed the administration of intensified full doses CT combined with the adequate planned pre- or post-op dose of RT. The study is ongoing in order to accrue the foreseen number of patients.

**Multiple primary malignancies in patients with soft tissue sarcomas**

O. MERIMSKY, Y. KOLLENDER, J. ISSAKOV, J.BICKELS, G. FLUSSER & I. MELLER
*(Tel Aviv Sourasky Medical Center, Tel Aviv, Israel)*

*Introduction:* Modern cancer treatment has substantially increased the survival of patients with various malignancies. One of the late sequelae of a successful treatment is the development of a second malignant tumor. However, in many cases of second primary cancers, exposure to chemotherapy or radiation therapy is not evident, and it should be postulated that the putative mechanism for the development of the second tumor is different.
*Methods:* Retrospective search of data files of 610 patients with soft tissue or bone sarcomas were treated by our team of Musculoskeletal Oncology from January 1995 through December 1999 was performed.
*Results:* Out of 375 patients with STS, 28 (7.5%) developed other malignant neoplasms either before or after its diagnosis. STS as the *first* tumor occurred in 14 patients. The *second* tumor types included mainly STS and renal cell carcinoma. The interval of time between the diagnosis of the STS and the second malignancy was 0–21 years. Three patients developed a *third* primary tumor within 0–3 years after the diagnosis of the second tumor. The median overall survival was >78 months. Fourteen patients had a first primary tumor *other* than STS. The second tumors (mainly STS) appeared within 0–27 years. The median overall survival of the 14 patients in this group from the diagnosis of the first tumor was >102 months.
*Conclusions:* The phenomenon of two or three primary neoplasms in patients in whom one of the tumors was STS occurs in a rate of 7.5% — a significantly higher rate than the occurrence of STS among the general cancer population (1%). Most of the cases occur incidentally. The clinical implications are the need to search for an occult second primary in patients with STS, as an integral part of their follow-up. It is especially true in patients with primary MFH who show a risk for developing a renal cell carcinoma.

**Neoadjuvant chemotherapy and preop irradiation of IIB soft tissue sarcomas**

M. HIZ, T. ÖGÜT, I. YÜCEL, N. MANDEL, F. DINÇBAS & S. DERVISOGLU
*(Cerrahpasa Faculty of Medicine, Orthopaedic Oncology Division, Istanbul, Turkey)*

*Aim:* Neoadjuvant chemotherapy and preoperative irradiation prior to wide excision of IIB soft tissue sarcoma of the extremities were applied to prevent the systemic metastasis and decrease the local recurrence rate.
*Subjects and method:* Between October 1989 and October 2000, 42 patients (29 male, 13 female) with a mean age of 40.7 (14–65) were treated with neoadjuvant protocol and all received wide local excision with uncontaminated margins. All patients were operated by the same surgeon. Histological distribution was 28 synovial sarcoma, nine malignant fibrous histiocytoma (MFH), two liposarcoma, two malignant mesenchymal tumor, one leiomyosarcoma. Localisations were: thigh 22, poplitea five, cruris six, knee one, foot four, upper extremity three, axilla one. Epirubicin 100 $mg/m^2$ and Iphosphamide 4 $\mu m/m^2$ with Mesna were applied to patients at three cycles with 3-week intervals together with 5000 cGy preoperative irradiation. A shrinkage with an average diameter of 1.3 cm was obtained. Wide excision was applied after the third cycle of chemotherapy and the same chemotherapy regimen was continued three more postoperative cycles regardless of the necrosis rate of the tumor. Mean follow-up was 32 (5–98) months.
*Results:* Oncological result was 23 NED, seven AWD, 12 DOD of the mean 32-month follow-up. Local recurrence rate was 4/42. One patient had skeletal and one had brain metastasis. All recurrent patients developed lung metastasis also. All recurrent patients were amputated and salvage chemotherapy was given, but all died at mean 8 months (6–11). Of the 19 patients who developed lung metastasis, seven received metastasectomy. Four of them are DOD.
*Conclusion:* Neoadjuvant chemotherapy and irradiation provided safe wide excision of huge IIB STS with 72% overall survival and 9.5% local recurrence at nearly 3 years follow-up.

**The value of MRI in determining the presence of residual tumour after the 'whoops' procedure**

R.J. GRIMER, A.M. DAVIES, A. MEHR, N. EVANS & P.B. PYNSENT
*(The Royal Orthopaedic Hospital Oncology Service, Birmingham, UK)*

*Aim:* Inadvertent excision of lumps which turn out to be soft tissue sarcomas is still unfortunately quite common. It is known as the 'whoops' procedure. Determining whether there is residual disease is key to deciding subsequent management. The value of MRI has been assessed.
*Method:* All new patients referred to our unit with a potential diagnosis of a 'whoops' lesion were routinely reassessed with MRI 6 weeks after the initial operation. Notwithstanding the result of the scan, all patients underwent a further wide excision of the involved area shortly after the MRI. The scans of these patients have been reviewed and classified into positive, equivocal or negative. These results have been compared with the histological assessment of the re-excision specimen to determine the accuracy of MRI in predicting the presence of residual tumour.
*Results:* Of 887 patients with newly diagnosed soft tissue sarcomas seen in an 8-year period, 140 (11%) had previously had a 'whoops' procedure. Of these, 111 had re-evaluation MRI scans and had also undergone a further re-excision. There was residual tumour in 63 (57%) patients, whilst 48 (43%) had no residual tumour.

| MRI prediction | Tumour present | Tumour absent |
|---|---|---|
| Positive ($n$=41) | 38 | 3 |
| Equivocal ($n$=7) | 4 | 3 |
| Negative ($n$=63) | 21 | 42 |

The sensitivity of MRI in predicting tumour was 64%, but specificity was 93%. Positive predictive value was 93% and negative predictive value 67%. Overall accuracy was 77%.
*Conclusion:* MRI is useful in identifying residual tumour after a whoops procedure but a negative result by no means excludes it. Re-excision remains essential despite the MRI results in most cases to ensure tumour clearance. Preventing the 'whoops' procedure is clearly the best option of all!

### Session 5: Metastatic disease: surgery vs radiotherapy in relation to life expectancy

**Primary metastatic osteosarcoma: surgery and response of the primary have major prognostic importance.**

S. BIELACK, S. FLEGE, M. KEVRIC, D. BRANSCHEID, S. LANG, H. JÜRGENS & A. ZOUBEK (FOR THE COOPERATIVE OSTEOSARCOMA STUDY GROUP COSS)
*(Department of Pediatric Hematology/Oncology, University Muenster, Muenster, Germany)*

*Aim:* To define factors which might predict survival in primary metastatic osteosarcoma.
Patients and methods: All patients with previously untreated (except primary surgery) high-grade osteosarcoma of the extremity or trunk registered into a neoadjuvant COSS study before seven of 98 who presented with primary metastases were included into an analysis of patient-, tumor-, and treatment-related factors and outcome.
*Results:* Two hundred and eleven patients with primary metastatic osteosarcoma (histologically verified 128, proven by progression 54, diagnostic imaging only 29). After a median follow-up of 1.6 years for all patients and 3.7 years for survivors, 80 patients were still alive, 34 of these in continuously complete surgical remission. Actuarial overall survival at 5 years (5 yr OS) was 32%. (When the 29 patients with abnormal diagnostic imaging as the only suggestion of metastases were not included, 5 yr OS was only 27%.) Among pretreatment variables, site of the primary tumor (189 limb: 5 yr OS 34%; 22 trunk: 13%) and of the metastases (155 confined to the lungs: 5 yr OS 38%; 56 extrapulmonary involvement: 11%) were the only identified factors with prognostic impact. The 71 patients whose primary tumors responded well to chemotherapy had a superior outcome (5 yr OS 53%) compared to the 62 with poor response (24%). Ninety-three patients with a macroscopically complete surgical remission of both the primary and all metastases had a 5 yr OS of 51%, while almost none of the patients with any remaining focus of proven tumor survived. 5 yr OS was 65% for 46 patients with both a good response and complete surgery.
*Conclusion:* In osteosarcoma, all efforts must be taken to remove both the primary tumor as well as all metastasis. Prognosis is improved in those patients who achieve a good tumor response to chemotherapy.

Supported by Deutsche Krebshilfe

**Postoperative radiotherapy on indication is effective treatment of cemented hemiarthroplasty in pathological fractures of the proximal femur**

P.D.S. DIJKSTRA, T. WIGGERS & H. BOXMA
*(Orthopedic Surgery, Leiden University Medical Centre, Leiden; Surgical Oncology, Daniel Den Hoed Cancer Center, Rotterdam; Surgery, Medical Center Rijnmond-Zuid, Rotterdam, The Netherlands)*

The routine use of radiotherapy after surgical fixation of a pathological fracture is still controversial. In this study the indication for radiotherapy after surgery was persistent or recurrence of pain postoperatively, and metastatic cortical lesion close to tip of the stem. A retrospective analysis of pain relief, mobilisation, complications, radiotherapy after surgery for pathological fractures of the proximal femur due to bone metastases in 50 patients was carried out. Breast carcinoma was the primary tumor in the majority of the patients. These patients with 52 fractures, 40 actual and 12 impending, were treated with cemented hemiarthroplasty. The patient survival rate was 55% after 6 months. Pain relief was achieved in 90% of the patients. Forty-four patients (85%) were able to walk in 7 days postoperatively. There was one local complication. In five cases the endoprothesis failed (range 1–55 months); luxation, refracture ($n$=2), and loosening ($n$=2). Nine patients received postoperative radiotherapy (median 24 Gy, range 20–30 Gy), four within 8 weeks after surgery, and four of these nine patients had failure of endoprothesis. If pain recurrence was the indication for postoperative radiotherapy all patients sustained pain reduction. Two-thirds of the patients received preoperative radiotherapy (median 24 Gy, range 8–30 Gy), in 12% of these patients radiotherapy was also performed after surgery. Preoperative radiotherapy had no correlation to failure of device or pain recurrence. In conclusion, our results show that *not* routinely performing radiotherapy (24 Gy) after cemented hemiarthroplasty for the treatment of pathological fractures is a safe method to improve quality of life. There is a need for a prospective randomised study about the use of routine postoperative radiotherapy.

**Metastatic disease after primary sarcoma and carcinoma: a need for a standardized surgical therapy regimen (the University of Muenster experience)**

N.J. LINDNER, G. GOSHEGER, T. OZAKI, C. HOFFMANN, C. GEBERT, R. RÖDL, F. BÖTTNER & W. WINKELMANN
*(University of Münster, Germany)*

*Aim:* The prognosis of metastatic disease in bone is poor, but a proportion of patients may survive for several months or even years and will require active treatment. The aim of this study was to evaluate indications, various strategies of surgical treatment of bone metastases after sarcoma and carcinoma and their outcome and influence on the course of the disease.
*Methods:* In a retrospective study we analyzed 184 patients with bone metastases. Parameters were primary tumor, localization, type of metastases, indication for operative treatment, type of surgery performed and pre- and postoperative adjuvant treatment. Complications and results of treatment including pain, mobility and quality and duration of life were evaluated.
*Results:* In 157 patients one procedure was indicated, 13 patients had two operations. We found a high rate of primary unknown tumors (50, 27.2%) and osteolytic metastases (159, 86.4%). Most frequent indications were pain (129, 70%) prophylactic fixation (75, 40.7%) and fracture (46, 25%). Operations were posterior stabilization (39, 24.8%), tumor prosthesis (35, 22.2%), osteosynthesis with curettage and cementation (19, 12.1%) and curettage and cementation (19, 12.1%). Adjuvant regimens like pain-, radio- and chemotherapy were performed pre- and postoperatively. Preoperatively 41 (26.1%) patients received radiotherapy and 17 (10.8%) chemotherapy. Postoperatively 64 (40.7%) patients received radiotherapy, 48 (30.5%) analgesic therapy and 25 (15.9%) chemotherapy. Complications delaying postoperative recovery were high and found in 52 (33.1%) patients. Clinical mortality was 4.4% (seven). Primary skeletal stability was found in 116 (73.8%) cases, mildly impaired function of the affected extremity in 93 (59.2%) and 73 (46.4%) were completely pain free.
*Conclusions:* With a rational standardized surgical strategy in combination with adjuvant treatment most patients get an acceptable stability and function of their extremity or spine and reach a better physiological and psychological constitution as well as local disease control.

**The effectiveness of surveillance programmes in patients with sarcoma**

R.J. REES, L. JEYS, P. COOL & R. GRIMER
*(Royal Orthopaedic Hospital, Birmingham, UK)*

*Purpose:* To assess the efficacy of the current surveillance programme for patients with sarcoma. Surveillance programs are important to enable both local recurrence and metastatic disease to be picked up earlier, which could improve survival. The medical literature is lacking with regards specific guidelines for surveillance. Whooley *et al.*[1] reported their method of surveillance in 176 patients with soft tissue sarcoma.
*Methods:* We undertook a prospective analysis of all patients with sarcoma treated between 1990 and 1995.The patients routinely enter a surveillance programme which consists of regular clinical evaluation, CXR and radiological imaging.
*Results:* Seven hundred and twenty-six cases of sarcoma with mean follow up 8.4 years (range 6.2–11.3). Local recurrence occurred in 14% of cases and 34% developed metastases. The cumulative survival at 10 years was 59% (5 years 66%). Forty-six percent of the deaths were directly attributable to metastases. Clinical evaluation and CXR were found to be valuable tools for detecting local recurrence and metastases. The role of MRI and CT in surveillance was unclear.
*Conclusion:* Surveillance programmes are valuable in the management of patients with sarcoma but their precise role needs to be well defined.

**Bone metastases in breast carcinoma: therapy and prognosis**

H.R. DÜRR, P.E. MÜLLER, T. LENZ, V. JANSSON & H.J. REFIOR
*(Department of Orthopedics, Ludwig-Maximilians-University Munich, Germany)*

*Aim of the study:* Bone metastases are commonly found in breast cancer patients. In this retrospective study, we evaluated the effect of surgical therapy on a series of 70 patients with breast cancer who were surgically treated for metastasis of the bone.
*Methods:* The patients' age, treatment of the primary tumour, time of appearance of bone and visceral metastases, clinical symptoms, surgical treatment, complications, and survival were reviewed. The most common locations of bone metastasis were the spine ($n$=29), and the proximal femur ($n$=27). Pathological fractures were found in the spines of 17 patients and in the extremities of 22 patients. Ten patients showed neurological impairment. At presentation, 19 patients had a singular osseous lesion, 19 patients showed multiple bone lesions, and 32 patients had additional visceral involvement. The surgical procedures included 60 palliative procedures, six radical resections and four biopsies. Presently, 56 patients have died of their disease. In the 14 surviving patients, the mean observation period is 35.6±40.1 months.
*Results:* No patient had local recurrences or local tumour progression. Of the six patients with radically resected solitary bone lesions, five developed systemic progression of the disease. Of the 19 patients with presumably solitary bone lesions, five appear to be free of tumour at present. Of the 19 patients with multiple bone lesions and initially no visceral tumour spread, only two patients are still alive. Of the 32 patients with additional visceral metastases at surgery, four are alive with the disease. For the entire group, the survival rate was 59% after 1 year, 37% after 2 years, 13% after 5 years and 7% after 10 years. The only two independent factors that were associated with survival were the extent of the disease and the duration of symptoms due to bone metastasis.
*Conclusions:* Orthopaedic surgery in patients with bone metastases secondary to breast cancer and bone metastases should be confined to those patients whose impending or actual fractures or neurological complications force surgical therapy. The comparably long survival rate in this group of patients and the proven prognostic factors associated with a particular patient should be taken into consideration when deciding which intervention is appropriate.

**Resection and reconstruction versus transfocal internal stabilization in the treatment of metastatic bone diseases**

I. ANTAL, T. L_RINCZ, M. PATCZAI, A. SÁRVÁRY, P. SZÉPHALMI & M. SZENDR_
*(Semmelweis University of Budapest, Budapest, Humgary)*

*Purpose:* The impact of two different methods, i.e., resection and reconstruction versus transfocal internal stabilization of bone metastases was studied in 209 patients concerning the survival, life quality and local complications.
*Material and methods:* Three hundred and thirty-eight patients with bone metastases were treated in two Institutions during the period from 01/1990 to 01/2000. The minimum follow-up was 12 months. 129 patients were excluded for different reasons. One hundred and nine patients (Group I) were treated with complete resection of the metastasis followed by reconstruction (endoprotheses in 58 patients, bone grafts in two patients, blading and bone cement in 37 patients, resection only in 12 patients) and 100 patients (Group II) with internal transfocal stabilization (curettage, bone cement and blading; intramedullary (interlocking-, Gamma- or Ender-nailing) fixation and/or bone cementing. The patients of the two differently treated groups were matched according the general condition (Karnofsky), site of the primary tumor (lung, breast, kidney, prostate and others). For the statistical analysis Fisher's exact-test, the *t*-test, the Kaplan–Meier survival curve and the Cox–Mantel test were used.
*Results:* The survival differed significantly in the two groups: in Group I 46.6%, 1-year, 20.5%, 2-year, 10.7%, 3-year and 2.6%, 5-year survival rates were detected; versus 15.1%, 1-year, 4.04%, 2-year and 0% 3-year survival rates in Group II. Concerning the site of the primary tumors the mean survival in kidney metastases (17 months) was superior to the breast (11.3 months) and lung (6.7 months) metastases.
*Conclusions:* The type of surgery does not influence the final outcome of the disease. Resection and reconstruction is recommended in the early stage (solitary form) and translocal internal stabilisation in the more advenced stage (multiple form) of bony metastases. At correct indication, the rate of complications is low (group I, 8% and group II, 11%) and comparable in both groups.

**Treatment options in spine metastases of renal cell carcinoma**

A. GASBARRINI, S. BANDIERA, M. CAPPUCCIO, G. BARBANTI, F. DE IURE, G. PIZZA & S. BORIANI
*(Department of Orthopedics and Traumatology. Istituti Ortopedici Rizzoli, Bologna, Italy)*

*Aim:* Evaluation of the different surgical options (simple decompression and stabilization versus intralesional excision versus *en bloc* resection) in the treatment of spinal metastases from renal cell carcinoma (RCC).
*Methods:* Fifty cases in 44 patients with symptomatic spine metastases secondary to RCC were reviewed between 1974 and 2000. Eleven patients were submitted to radiation therapy only. In 16 cases a palliative procedure was adopted, in 18 cases a curettage and in five cases an *en bloc* resection (two were marginal while three were wide). Seventeen cases were operated on for neurological dysfunction, 27 for progressive pain. Radiation therapy (RTR) was performed after the surgical procedure in 32 cases, preoperative selective arterial embolization (SAE) in 11 cases, chemotherapy in five cases and immunotherapy (IL2) in 14 cases.
*Results:* All cases were followed for at least 6 months (6–69 months; average 15.3). The local recurrence percentage was none out of three when it was done with a wide margin. The mean survival was 5.8 months (min. 2; max. 14) in the 11 patients who were treated only with RTR, 13.5 months (min. 10; max. 16) in patients submitted to palliative surgery (six out of 16 died) and 29.8 months (min. 14; max. 69) in patients submitted to curettage (five out of 18 died). Of the five patients operated with *en bloc* resection only one died after 6 months.
*Conclusions*: The treatment of spine metastases of RCC is multidisciplinary: the eligibility of patient to *en bloc* resection both on the basis of general conditions and local tumor expansion is critical for the local control of the disease. If *en bloc* resection is not feasible, a combination of SAE, complete extracapsular intralesional excision, RTR and immunotherapy seems to be the most appropriate treatment.

**Life expectancy and surgery or radiotherapy for spinal metastases: proposal based on a surgical experience**

C. VILLAS
*(Department of Orthopaedics. University of Navarra, Pamplona, Spain)*

*Introduction:* Therapeutic decision making for patients with spinal metastases is difficult because of the influence that many factors have on results and risks of surgical treatment. Among these possible influencing factors, life expectancy may theoretically be a good one when trying to establish an algorithm. Nevertheless, in daily practice, we often miscalculate life expectancy in many patients. Although radiotherapy may bring effective improvement of pain and neurological impairment, it usually needs several days or weeks and frequently closes the door for future surgery. When spinal cord compression with neurological impairment exists, a few days of delaying decompression may be crucial for patient's recovery chance.
*Patients and methods:* From 1979 to1995 we have operated on 121 patients complaining of untreatable pain and/or spinal cord compression. Evaluation included symptoms relief and patients' survival.
*Results:* Even patients with short life expectancy benefited from surgery which proved to be very effective in relieving pain and improving neurological function. Sixty-one patients received chemotherapy and/or radiotherapy before surgery with no satisfactory improvement of their complaints. Only 18 out of the 106 patients with untreatable pain remained in a drug-dependent situation after surgery; five morphine-dependent patients referred no improvement after surgery. Seventy-three (80%) out of the 91 patients with neurological impairment in our series were unable to walk before surgery, and only 22 were not able to walk after surgery (76% of recovery). Survival was longer than one year in 22% of the patients. *Discussion:* For patients with short life expectancy, radiotherapy may reasonably be the first therapeutic step when the patient has been informed and is aware of (and accepts) the risk of suffering a possible paraplegia. In deciding treatment, it is important to remember that time is relative and what may seem like a short period of time from the doctors perspective, may seem an eternity for a suffering patient.

### Session 6: Free papers

**Axial osteosarcoma: local surgical control is still of paramount importance in the age of interdisciplinary therapy**

S. FLEGE, M. KEVRIC, V. EWERBECK, U. EXNER, U. HEISE, A. HILLMANN, H. JÜRGENS, R. KOTZ, R. WINDHAGER, W. WINKELMANN & S. BIELACK (FOR THE COOPERATIVE OSTEOSARCOMA STUDY GROUP COSS)
*(Department of Pediatric Hematology/Oncology, University of Muenster, Muenster, Germany)*

*Aim:* Osteosarcoma of the trunk is rare. We set out to delineate the role of surgery in this setting by evaluating a comparatively large cohort of patients with localized axial disease.
Patients and methods: All patients with previously untreated (except primary surgery) high-grade osteosarcoma of the axial skeleton registered into a neoadjuvant COSS study before seven of 98 were evaluated for local control and survival. All protocols called for intensive polychemotherapy and — whenever feasible — complete surgery.
*Results:* Eighty-five patients with localized axial osteosarcoma were identified (49 male, 36 female; median age 19 years (range 5–62); 73 osteosarcoma as a primary, 12 as a secondary malignancy; tumor sites: pelvis 61, rib 10, spine seven, scapula five, clavicle two). After a median follow-up MFU of 2.0 years for all patients and 3.1 years (0.03–17.4) for survivors, 43 patients were still alive, for an actuarial 5-year survival rate (5 yr OS) of 40%. Fifty-three patients (62%) achieved a macroscopically complete surgical remission. Thirty of these survived (5 yr OS 53%). Forty-one achieved permanent local surgical control (48% of all 85 patients and 77% of the 53 who achieved a local surgical remission). Twenty-nine of the 41 survived (MFU 5.3 years, 5 yr OS 70%). By comparison, 5 yr OS was only 4% for 42 patients without permanent local surgical control, with only one patient alive past 5 years (two patients without sufficient information on local disease status).
*Conclusion:* Patients with localized axial osteosarcoma in whom permanent local control can be established may enjoy an outcome similar to that of patients with limb tumors. There is a dire need for alternative strategies for those who fail locally.
Supported by Deutsche Krebshilfe

**A comparison between sporadic and neurofibromatosis-associated malignant peripheral nerve-sheath tumours**

V. PRASAD[1], D.E. PORTER[1], R. BIRCHE[2], R.J. GRIMER[1], S.R. CARTER[1] & R.M. TILLMAN[1]
*([1]Royal Orthopaedic Hospital Oncology Service, Woodlands, Birmingham; [2]Peripheral Nerve Injury Unit, Royal National Orthopaedic Hospital, Stanmore, London, UK)*

*Introduction:* Malignant peripheral nerve sheath tumours (MPNSTs) constitute 10% of soft tissue sarcomas. A significant proportion arise in neurofibromatosis type 1 (NF1). Several publications have compared MPNST survival in sporadic and NF1 patients, without consensus on whether NF1 is an independent factor for poor prognosis.
*Methods:* Clinical and histological data from 135 proven MPNSTs were analysed from two national centres for soft tissue tumour surgery diagnosed from 1979 to 2000. One hundred and twenty-nine patients had follow-up data from 6 months to 21 years. Thirty-five were from patients with NF1. Local treatment involved surgery in surgery in 95%, radiotherapy in 44% and chemotherapy in 21%.
*Results:* NF1 patients were younger than those with sporadic tumours (median age 26 vs. 53 years, $P<0.001$). Overall MPNST survival was almost identical to that in soft tissue sarcomas as a whole, but was worse in NF1 than in sporadic tumours (33 vs. 72% at 30 months ($P<0.01$), 17 vs. 39% at 60 months, 6 vs. 21% at 120 months). A trend towards shorter time to local recurrence was seen in NF1, but not time to metastasis. Superficial tumours gave improved prognosis. Tumour volume over 100 ml was associated with worse survival (46 vs. 91% at 30 months, $P<0.02$), as was histological grade (80% high grade vs. 25% low grade at 60 months, $P<0.01$). In terms of location, a non-significant over-representation of NF1 MPNSTs in the sciatic and brachial plexii was identified.
*Discussion:* NF1 and sporadic MPNSTs exhibited no difference in depth or tumour volume profile, although NF1 tended towards higher grade. Analysis of survival in only high-grade tumours, however, still resulted in a significant survival disadvantage in NF1 (33 vs. 70% at 30 months, $P<0.01$). Removal or brachial and sciatic plexus tumours from analysis did not affect survivorship profiles in NF1 and sporadic MPNSTs.
*Conclusions:* Grade, volume and tumour depth correlate with survival; only seven of 45 patients with deep high-grade tumours over 100 ml volume were observed to survive beyond 2 years. MPNST survival is worse in NF1 than sporadic tumours. Grade, depth, site and volume differences could not explain this disadvantage.

**Extra-abdominal desmoid tumor: a new treatment modality including interferon-alpha and tretinoin**

A. LEITHNER, R. WINDHAGER, B. SCHNACK, T. KATTERSCHAFKA, C. WILTSCHKE, G. AMANN, R. KOTZ & C.C. ZIELINSKI
*(Karl Franzens University of Graz, Graz, Austria)*

*Aim of the study:* The postsurgical treatment of extra-abdominal desmoid tumors (also called aggressive fibromatosis) is uncertain. Therefore, a retrospective study was undertaken, to evaluate the authors experiences with a new treatment regime including interferon- (IFN-±tretinoin.
*Methods:* Thirteen patients with extra-abdominal desmoid tumors and a median age of 32 years (range 15–73) received IFN-± and tretinoin in order to prevent recurrence or to stabilize progressive disease.
*Results:* After a mean observation period of 27±15 months (mean±standard deviation) under treatment with IFN-±, local control was seen in 11 of 13 patients (85%). Seven patients had no evidence of disease at a mean disease-free interval of 22±18 months; in two patients progressive disease occurred after only 7 and 9 months, respectively, of observation. In another four patients, progression of the desmoid tumor was stabilized.
*Conclusions:* A literature review and data of this retrospective, nonrandomized study on therapy with IFN-±tretinoin suggest that such treatment may be affective in prolonging the disease-free interval or to stabilize progressive disease. This regimen should be considered as another nonsurgical treatment alternative.

**Neoadjuvant chemotherapy for non-metastatic osteosarcoma of the extremity: results of the protocol IOR/OS-4**

S. FERRARI, M. MERCURI, F. BERTONI, P. PICCI, A. LONGHI, M. MANFRINI, R. CAPANNA*, A. TIENGHI°, A. BRACH DEL PREVER+, A. COMANDONE# & G. BACCI
*Dipartimento di Oncologia Muscolo-Scheletica Istituto Ortopedico Rizzoli; * C.T.O. Firenze; ** Oncologia Medica Ravenna; *** Clinica Pediatrica Università di Torino; **** Oncologia Medica Ospedale Gradenigo Torino. ITALY.*

Our previous experiences (Bacci, 1993) had shown that patients with nonmetastatic osteosarcoma having a poor histological response to a primary chemotherapy with methotrexate (MTX), cisplatin (CDP), and doxorubicin (ADM) could benefit from the addition of ifosfamide (IFO) in the postoperative phase of treatment. From January 1993 to July 1995 a neoadjuvant chemotherapy protocol (IOR/OS-4 ) based on the preoperative use of IFO in all patients, added to CDP, ADM and MTX was carried out at our institution. Primary chemotherapy consisted of MTX 12 $g/m^2$ (weeks 0–4), CDP (120 $mg/m^2$) plus ADM (60 $mg/m^2$) on week 1, IFO (6 $g/m^2$) plus CDP (120 $mg/m^2$) on week 5, and IFO (6 $g/m^2$) plus ADM (60 $mg/m^2$) on week 8. Postoperatively, patients received MTX 12 $g/m^2$ (weeks 15, 24, 33, 42), ADM 90 $mg/m^2$ (weeks 11, 21, 30, 39), CDP 120 $mg/m^2$ (weeks 16, 25, 34), and IFO 10 $g/m^2$ (weeks 19, 28, 37). One hundred and thirty-three patients (median age 16, 3–40) with non-metastatic osteosarcoma of the extremity entered the study. Two patients died during the preoperative phase (one suicide, one toxic death). One hundred and thirty-one patients underwent surgery: limb salvage surgery in 123 (94%) patients; three rotationplasties, and five amputations. During the postoperative phase two chemotherapy-related deaths were registered (acute renal failure, and veno-occlusive disease). With a median follow up of 6 years, 75 (56%) patients were event-free; 54 (41%) relapsed with distant (44, 33%) or local (10, 7.5%) recurrence. Of the 54 patients who relapsed, 12 (22%) were NED II, four (7.5%) were AWD, and 38 (70.5%) died of disease. The 7-year cumulative probability of EFS and OS were 56% (95% CI 48–65), and 69% (95% CI 59–78), respectively. In the previous Rizzoli study (IOR/OS-3) based on the use of IFO only in poor responder patients, the 7-year EFS and OS were 54% (95% CI 44–64) and 61% (95% CI 51–71), respectively; the local recurrence rate was 3%. In non-metastatic osteosarcoma of the extremity, the use of MTX, CDP, ADM and IFO since the preoperative phase does not seem to offer advantages compared to the use of MTX, CDP and ADM with IFO delivered in the postoperative phase and in poor responder patients only.

**Urinary excretion of pyridinium crosslinks in patients with osteosarcoma**

S. FERRARI, C. ZOLEZZI, M.C. FASANO, L. PRATELLI L & G. BACCI
*(Dipartimento di Oncologia Muscolo-Scheletrica, Laboratorio Analisi, Istituto Ortopedico Rizzoli, Bologna, Italy)*

Pyridinium crosslinks (Pyr, D-Pyr), are molecules of collagenic origin currently considered markers for the diagnosis of bone metastases. Disturbances in bone remodelling in patients with primary bone tumors have been little investigated. The aim of the present study was to investigate the urinary excretion of Pyr, D - Pyr, alkaline phosphatase (AP) and and serum osteocalcin (OC), in patients with osteosarcoma of the extremity. Thirty-six patients (median age 14 years, 7–22) with osteosarcoma of the extremity (six metastatic at presentation) entered the study. A control population of age- and sex-mached healthy individuals was studied. Urinary excretion of Pyr, D -Pyr were measured on fasting urine specimens, corrected for creatine excretion (Ucr), and expressed as pM/μM UCr. At the same time of urine collection, blood samples were taken for measurement of AP, and OC. The urinary excretion of Pyr and D -Pyr were significantly (*P* 0.005, *P* 0.003) higher in patients with osteosarcoma (481.5±346, 82.7±59) than in control population (231±134, 38.2 ± 22.5). No differences were found according to the osteosarcoma histological subtypes, or to the presence of metastases. Tumor volume did not correlate with urinary excretion of Pyr and D-Pyr. A significantly higher excretion of Pyr (*P*=0.057) and D-Pyr (*P*=0.02) was found in patients who relapsed (Pyr 578.1±374, D-Pyr 96.8±65.3) compared to those who remained continuously free of disease (Pyr 304.5±134, D-Pyr 58.1±22). A highly significant correlation was found with AP, whereas no correlation was found with OC. The serum level of OC was significantly (*P* 0.0001) lower in patients with osteosarcoma (4.8±5.3 ng/ml) than in control population (20.3±7.6 ng/ml). The present study showed significant abnormalities of the urinary excretion of pyridinium crosslinks in osteosarcoma patients. Their possible use to monitor treatment can be proposed. The possible relation between baseline urinary excretion of pyridinium crosslinks and biological tumor aggressiveness observed in the present study require further investigations.

**Rehabilitation after surgery of tumors of the foot**

AXEL HILLMANN[1], CAROLA BERTSCH[1], GEORG GOSHEGER[1], DIETER ROSENBAUM[2] & WINFRIED WINKELMANN[1]
*([1]Orthopaedic Department, [2]Movement Analysis Lab, University of Muenster, Muenster, Germany)*

*Introduction:* Primary malignant tumors of the foot are rare. Complete resection is recommendable according to the Enneking classification. When the tumor is destructive an invasive amputation is required in many cases. In primary bone or soft tissue tumors a partial resection of the foot is possible, when the tumor shows a good response to adjuvant chemotherapy and radiation. In the present study the functional outcome has been evaluated by plantar pressure measurements.
*Material and Methods:* Six patients with a high grade malignant tumor of the foot (2nd metatarsal, $n$=3; 4th and 5th ray, $n$=1; Syme amputation, $n$=1) were clinically and functionally examined. Plantar pressure measurements were taken for barefoot walking and inside the shoe with capacitive pressure distribution platform and insoles (EMED & PEDAR, Novel, Munich). Several steps during full gait were selected and averaged to present a typical loading pattern. Foot loading parameters were determined for different regions of the foot and compared with the contralateral side.
*Results:* Walking ability depended in general on the degree and location of the resection. The patients with resection of the 2nd ray showed an increased loading of the midfoot and fore foot with decreased load under the toes. Both of them had a good foot function with high mobility. The patient with a resection of 4th and 5th ray had a decreased area and force under midfoot and forefoot, with a decrease in peak pressure under the toes as well. Peak pressure under the forefoot, however, increased clearly. A resection of the calcaneus led to decreased loads under the fore foot, hallux and toes. An amputation in the Chopart joint line was associated with an almost similar loading situation of the stump compared with the contralateral hind foot. Wearing an individual forefoot prosthesis led to increased loading of the stump.
*Discussion:* The foot loading parameters change after partial resection of the foot due to a primary bone tumor. In the case of ray resection good functional results and loading parameters similar to a normal foot can be observed. In shoe pressure measurements are a valuable tool to evaluate the effect of orthotics.

**Treatment of aneurysmal bone cyst by direct injection of ethibloc in children**

E. MASCARD, C. ADAMSBAUM, G. KALIFA & J. DUBOUSSET
*(Pediatric Surgery and Radiology Departments, Hospital Saint-Vincent de Paul, Paris, France)*

Aneurysmal bone cysts (ABC) are benign but sometimes very aggressive lesions. The accepted treatments are aggressive bone curettage or marginal resection. Ethibloc is a fibrogenic and thrombogenic agent used for vascular embolisation. Our purpose was to report the results of treatment of ABC by Ethibloc. From 1991 to 2000, 16 patients with aneurysmal bone cyst were treated by direct injection of Ethibloc. There were 12 females and four males, aged 2–14 years (mean 8 years). The diagnosis was always assessed by open biopsy. Six patients had various previous surgical treatments. Locations of the lesions were the proximal femur (four), the proximal humerus (three), the acetabulum (two), the clavicle (two), the distal fibula (two), the distal humerus (one), the distal ulna (one), and the second metacarpal (one). All injections were performed under general anesthesia, with control by image intensifier or CT scan. Before Ethibloc injection, opacification of the cyst was achieved by contrast medium to look for vascular drainage. Results were retrospectively assessed at 3-year mean follow-up (6 months to 7 years). Immediate complications were three cutaneous fistulae that healed spontaneously, in which one underwent a scar excision for cosmetic reasons. Most of the patients developed fever and inflammatory local reaction after the injection. There was no deep infection, no pulmonary embolism, and no malignant degeneration. In two cases, the injection had no effect on the ABC, requiring surgical excision, and in one case the remaining cyst had to be curetted. All other 13 patients had complete healing or ossification of the ABC. In conclusion, direct Ethibloc injection is a minimally aggressive but effective treatment of ABC. Because of the possible intense inflammatory reaction, we do not recommend that treatment in spinal or distal (hand and foot) lesions.

**Giant cell tumour of bone in the elderly**

V. PRASAD, D.E. PORTER, N. EVANS, R.J. GRIMER, S.R. CARTER & R.M. TILLMAN
*(Royal Orthopaedic Hospital Oncology Service, Birmingham, UK)*

*Introduction and aims:* Giant cell tumour of bone (GCT) occurs in a characteristic age distribution, approximately 75% arising in the third and fourth decades of life, the incidence diminishing rapidly thereafter. In fact, a literature review encompassing 473 GCTs records only eight cases diagnosed beyond age 70. Although GCT characteristics are described in the skeletally immature, no detailed analysis has been undertaken in the elderly, which was the purpose of this study.
*Methods and results:* From 1981 to 2000, 334 histologically confirmed primary GCTs were treated in our Unit. Five presented in patients over age 70, including, perhaps, the oldest recorded in the literature. All were referred from other hospitals, four with a provisional clinical diagnosis of metastatic bone disease, probably because of their age.

| Age (years) | Site | Pain swelling | Eccentric | Well defined margins | Treatment |
|---|---|---|---|---|---|
| 73 | Distal radius | Yes | Yes | Yes | Curettage |
| 74 | Fibular head | Yes | Yes | Yes | Curettage |
| 72 | Distal femur | Yes | Yes | No | Curettage& cement |
| 75 | Proximal femur | Yes | No | No | Endoprosthesis |
| 78 | Humeral head | Yes | No | No | Died prior to surgery |

Clinical and radiographic review classified two cases as active benign GCTs at typical sites, two as active aggressive GCTs at atypical sites, and one with intermediate features. Investigations (including biopsy) and treatment options were identical to those in younger patients, and successful, although one patient died of lobar pneumonia prior to surgery.
*Discussion and conclusions:* GCTs do occur in the elderly (although perhaps not beyond age 80). Biopsy is mandatory to exclude metastatic or primary malignant bone disease. Treatment as for younger patients is successful.

**Chondroblastoma of bone**

R. SUNEJA, M. BELTHUR, N. DESHMUKH, R.J. GRIMER, S. CARTER & R.M. TILLMAN
*(Royal Orthopaedic Hospital Oncology Service, Birmingham, UK)*

*Introduction:* Chondroblastoma is generally accepted as a benign neoplasm, which largely manifests in adolescence during the period of active epiphyseal plate growth. The treatment modalities for this condition are quite variable and the rates of recurrence following them controversial.
*Aims:* This study was carried out with the aim to evaluate the rates of recurrence following the intralesional curettage technique and evaluate the functional outcome objectively using the Enneking's functional evaluation scoring system. We have also attempted to define factors associated with more aggressive tumour behaviour.
*Methods:* This is a retrospective study of 70 patients with chondroblastoma treated over the past 27 years at our unit. The age at presentation, sex, anatomical site, signs and symptoms, operative procedure and post-operative complications were noted. The radiographs were reviewed for the location and size of the lesion, the amount of cortical destruction and the status of the adjacent epiphyseal plate. The histopathology slides were studied with special emphasis on the presence or absence of the aneurysmal bone component.
*Results:* Out of the 70 patients included initially, 53 had their primary treatment procedure performed at our unit in the form of an intra-lesional curettage, and were followed-up of at least 22 months, up to a maximum of 27 years. There was a histologically proven local recurrence in six out of these 53 cases (11.3%). Three patients underwent a second intralesional curettage procedure and had no further recurrences. Two patients had endoprosthetic replacement of the proximal humerus and one patient underwent a below knee amputation following aggressive local recurrences. One patient had the rare malignant metastatic chondroblastoma and died eventually. We were able to perform an objective assessment of the functional outcome in 40 patients (duration of follow-up 22–324 months) using Enneking's functional evaluation scoring system. The mean score was 94.1%.
*Conclusion:* We conclude that meticulous intralesional curettage without any additional procedure can achieve low rates of local recurrence and excellent long-term functional results.

**An algorithm for diagnosing non-traumatic bony lesions of the clavicle**

S.R. CARTER, S. MAIYA, P.B. PYNSENT, R.J. GRIMER & R.M. TILLMAN
*(Royal Orthopaedic Hospital Oncology Service, Birmingham, UK)*

*Aim:* To propose an algorithm for the diagnosis of bony lesions of the clavicle
*Introduction:* Non-traumatic lesions of the clavicle include infection, benign and malignant tumours and certain miscellaneous bizarre sclerotic lesions. The diagnosis of these conditions has traditionally been difficult not only due to the rarity of these lesions, but also due to the large variety of conditions that affect the bone and the problems encountered in imaging the clavicle.
*Materials and Methods:* We analysed over 102 patients with clavicular lesions referred to our unit over a 30-year period and looked at the case histories, investigation methods, diagnosis and outcome. We classified the lesions into four useful groups for clarity in analyses, viz: infections, benign tumours, malignant tumours and miscellaneous conditions.
*Results:* The distribution of lesions is similar to that seen in the long bones with the two ends of the bones being affected the most (67.4% in medial third and 21.9% in lateral third). Of the 102 cases, lesions were uniformly distributed with infection seen in 37(36.3%) cases, tumours in 39 (38.2%) and miscellaneous conditions seen in 26 (25.5%) of the patients. Infection was more commonly seen in the young and affected the medial third of the clavicle (94.6%). Tumours were seen in an older group and predominantly affected the lateral third (81%). Infection was more commonly sclerotic and expansile (77.1%), whereas tumours were lytic and destructive (88.2%). ESR, CRP and other blood parameters were not very helpful. Bone scans were useful only in detecting metastatic lesions and MRI scans were needed to localize these lesions and to pinpoint a site for the biopsy. An algorithm is presented to aid in the diagnosis of lesions of the clavicle.
*Conclusions:* This study has confirmed the difficulty in making a clinical and radiological diagnosis of lesions of the clavicle. Some patterns in presentation in terms of age, location, X-ray appearance and other imaging modalities are evident. But ultimately we advocate a low threshold for biopsy of these lesions to allow diagnosis.

**Is fruit a useful indicator of the size of an object?**

J.M. GRAY, M.T. KHAN, R.J. GRIMER & P.B. PYNSENT
*(Royal Orthopaedic Hospital Oncology Service, Birmingham, UK)*

*Background:* The use of inanimate objects is often used as a metaphor to describe the size or shape of pathological swellings, and it is our experience that soft tissue tumours are particularly described in this way (e.g., this lump is the size of a gape/apple/melon).
*Aims and methods:* This study aimed to assess the accuracy and therefore validity of this descriptive practice by questionnaire to orthopaedic specialists. Participants were asked to indicate the diameter of standard non-consumable items (tennis and golf balls) and consumables (fruit and vegetables) and also to estimate the size of a line drawing. Published sizes were found for objects.
*Results:* Current EEC sizes of fruit and vegetables were variable, most indicating only a minimum size. Golf balls also have a minimum standard and tennis balls a choice of standard sizes. Eighty-three questionnaires were completed. Estimated sizes of these objects varied widely. Estimation of the size of the line diagram was quite accurate.
*Conclusion:* Using fruit to describe the size of an object has no validity. Not only are there no standard sizes for fruit but orthopaedic surgeons perceptions of size also vary widely. Although less interesting in description the actual measurement of size should be the norm. Every surgeon should carry a tape measure in his/her pocket!

### ABSTRACTS FOR POSTERS

**C-ErbB-4 expression in limbs STS: correlation with neoadjuvant chemotherapy results**

O. MERIMSKY, J. ISSAKOV, J. BICKELS, Y. KOLLENDER, G. FLUSSER, S. VJACHESLAV, I. SCHWARTZ, M. INBAR, L. MELLER
*(The Tel-Aviv Sourasky Medical Center, Tel-Aviv, Israel)*

*Purpose*: ErbB-4 is a recently described member of the epidermal growth factor receptor (EGFR) family. Relatively little is known about the expression of erbB-4 in human tumors. In the present study we assessed the possible role of c-erbB-4 expression product as a tissue marker for STS, and its correlation with the response to chemotherapy.
*Patients*: The histological specimen of 29 patients with STS of a limb who had received preoperative doxorubicin-based chemotherapy were studied. The extent of tumor necrosis was evaluated histologically. Paraffin blocks of preoperative incisional biopsy were available for immune staining (avidin-biotin-peroxidase technique) from 29 patients, and blocks of the surgical specimen after pre-operative chemotherapy were available from 27.
*Results*: The objective response rate to preoperative chemotherapy was 34%. Wide resection of the tumor was feasible in 12 patients, marginal resection in 14 cases, amputation in 2 patients with disease progression, and no surgery in one case. The tumor necrosis was above 90% in 9 patients, 60–90% in 12, and less than 60% in 7 patients. It was found that an increase in C-erbB-4 expression was more common in cases with no response to chemotherapy, while no change of or decrease in C-erbB-4 was more common in responsive tumors (p=0.004). No correlation could be found between the degree of necrosis or the chemotherapeutic regimen and the change in expression of c-erbB-4. The median DFS was longer for patients with a decrease or no change in expression of C-erbB-4 than for patients with increased expression.
*Discussion*: It is believed that post chemotherapy new expression or no down-regulation of the erbB-4 molecule represents tumor aggressiveness and increased capability of growth and spread.

**Late complications after radiotherapy in ewing sarcoma**

ORLI_ D, SMERDELJ M, KOLUNDZI_ R, ORLI_ IVNA*
*(Department of Orthopaedic Surgery, School of Medicine, University of Zagreb, Croatia)*

Treatment of patients with Ewing's sarcoma in the last period is giving good results. Longer survival of patients has resulted sometimes in the observation of some late complications related to previous treatment. Our experiences with two patients were presented.

Radiotherapy and chemotherapy in a boy aged 12, in 1986 was applied. A healing process of the tumor was achieved with irradiation, but subsequently the extensive complications of applied irradiation developed. A year after irradiation atrophy and hypotrophy of the irradiated thigh, along with contracture of the hip joint and the knee joint developed. The leg was totally atrophic, shorter by some 10 cm with nonunion of a pathological fracture in the middle third of the femur. Amputation of the lower extremity because of quite miserable quality of life and completely afunctional leg 4 years and 10 months after was indicated. Histological examination of the extremity did not show any signs of a tumor. In all layers of the markedly atrophic tissue, i.e. the skin, subcutaneous fatty tissue, muscles and bone, signs of necrotic changes were presented with extensive calcifications and formation of scar tissue. Minimal or very slightly marked tissue regeneration was registered, while the osteoblasts were very faintly active. Similar changes were present in the blood and lymph vessels, causing edema and congestion.

Identical atrophic changes with good healing in early stage of partial stress fracture in the middle third of the femur were registered 15 years after irradiation of Ewing sarcoma in the young women. Today she is normal working mother with two children and excellent quality of life.

Late complications after contemporary radiotherapy in Ewing sarcoma are not clinical problem any more.

**Reimplantation of reirradiated tumour bone: advantages and pitfalls**

KHAN M., GRIMER R., PEAK D

*(Royal Orthopaedic Hospital Oncology Service. Birmingham, U.K)*

Limb salvage surgery is now the preferred method of treatment for malignant tumours of bone. It can be achieved by en-block resection of the bone followed b arthrodeses, endoprosthetic replacement or allograft reconstruction of the defect.

We have re-implanted the patient's bone for reconstruction of the defect after debulking the tumour and irradiating the bone. This method was used in ten patients with aggressive grade IIB sarcomas (osteosarcoma, Ewing's sarcoma) of the pelvis, humerus, tibia and metacarpal. Median overall survival of the patients was 24 months with a maximum survival of sixty-nine (69) months. Four patients were alive at the most recent follow-up. One of them is alive with metastatic disease and local recurrence while others remain free of disease. One patient has had pathological fracture through the irradiated bone that healed with conservative measures. One patient developed avascular necrosis of the femoral head and required resurfacing arthroplasty of the hip.

The pelvic sarcomas continue to be a challenge. Resection, extracorporal irradiation and re-implantation may offer some hope but it remains experimental. It should only be offered after a careful review of the other options for reconstruction. It is technically demanding and associated with a high incidence of complications.

**Result of limb salvage surgery (498 sarcomas in 25 years)**

DELEPINE GÉRARD[1], DELEPINE FABRICE[2], DELEPINE NICOLE[1]

*([1]Oncologic Ped. Sce Hop. Avicenne Bobigny, [2]Passage du bon pasteur, Rouen, France)*

*Methods*: From 1/1975 to 31/12/200, 498 bone sarcomas were treated by our multidisciplinary team. Average age of patients (p.): 27.1 years. Average size of the tumor (T): 13.1 cm. Histology: 231 osteosarcomas (OS), 118 chondrosarcomas, 104 Ewing (EW), 25 malignant histiocytofibromas, 12 fibrosarcomas and 8 miscellaneous. Locations: femur (203), innominate (98), tibia (86), humerus (60). Staging: III in 64. En-block resection was performed by the same surgeon. Histology of margins showed wide resection (295), marginal (185) and contaminated (18). Adjuvant therapy was adapted to age of p., histology and location. Postoperative RXT (35–50 grays) was administered in some adults with OS and EW, whose histological response to neoadjuvant chemotherapy (CT) was bad and resection marginal or contaminated.

*Results*: With a median FU of 11 y., 252 p. are alive without disease, 8 still receive treatment and 238 are dead from illness or complications of treatment. 35 local recurrences occurred, most of them (26) in p. who were referred after ineffective RXT or CT. Complications: 42 deep infections, 24 p. secondarily amputated. Functional results: At last FU, function excellent 52, good 35, fair 7%, poor 6%. It hanged mainly on size of T, could be altered by complications (infection) or RXT.

*Conclusion*: 1) L.S. is feasible in more than 95%, even for huge or fractured T. 2) The functional result is the best plae for L.S. 3) Risk of local recurrence for first hand H.G. sarcoma is only 2%. For low grade T or when no effective CT can be administered the risk of local recurrence increases. 4) RXT can help in local control for EW and mesenchymal chondrosarcoma but not significantly for others. As most of secondary amputation, and most poor function have been observed in irradiated p. RXT must be avoided. 5) What is the optimal timing for surgery? Preoperative CT is potentially dangerous for p. when insufficiently effective CT is administered for a too long period. In our practice, 1 month of preoperative CT for OS and 6 weeks for EW are enough to fulfil the needs of CT and surgery.

**Soft tissue reconstruction of megaprostheses using the mutars® – trevira-tube**

GEORG GOSHEGER*, AXEL HILLMANN*, HORST BÜRGER**, WINFRIED WINKELMANN*

*(Department of Orthopaedics* and Gerhard-Domagk-Institute of Pathology** University of Münster, Germany)*

In soft tissue reconstruction of megaprostheses the reattachment of soft tissue and joint capsules is essential. Sixty-nine megaprostheses were implanted and a trevira tube was applied to support reconstruction of the capsule and soft tissue. In the case of proximal femur replacement (33 patients), total femur replacement (five patients) and proximal humerus replacement (16 patients), the trevira tube allows for reconstruction of the capsule and refixation of the muscles and helps to minimize dislocation. In the case of a proximal tibia replacement (seven patients), arthrodesis of the knee (three patients), total knee replacement (two patients) and distal femur replacement (three patients), the trevira tube allows for attachment of muscle flaps and extensor tendons. Dislocation was observed in two of 54 patients only, who had proximal femur replacements. No dislocation was observed in patients with a total femur endoprosthesis or a proximal humerus endoprosthesis. Furthermore the trevira tube was used to attach the gastrocnemius muscle in patients with a proximal tibia endoprosthesis and to reattach the rotator cuff in patients with a proximal humerus prosthesis. In addition there was no significant increase in the rate of infection ($p = 0.46$; chi square test). The histopathologic findings in six patients showed a tissue ingrowth into the tube.

**Osteoid osteoma and osteoblastoma of the spine: experiences of 22 patients**

TOSHIFUMI OZAKI,[1] ULF LILJENQVIST,[1] AXEL HILLMANN,[1] HENRY HALM,[2] NORBERT LINDNER,[1] GEORG GOSHAGER,[1] AND WINFRIED WINKELMANN[1]

*(Department of Orthopaedics, Westfälische Wilhelms-University, Münster and [2] Department of Spine Surgery, Klinikum Neustadt, Neustadt, Germany)*

Osteoblastomas and osteoid osteomas of the spine are relatively rare bone forming tumors.
Between 1980 and 1999, nine patients with osteoid osteoma and 13 patients with osteoblastoma had surgical treatment for their tumors. Four tumors were in the cervical spine, six tumors were in thoracic spine, 10 tumors were in the lumbar spine, and two tumors were in the sacrum.
The average duration between onset of pain and surgery was 16.6 months in 12 patients treated in the 1980s and 8.6 months in 10 patients treated in the 1990s. Seventeen patients had scoliosis. In nine of 10 patients with magnetic resonance imaging scans, high signal intensity areas in the muscles and bone around the lesion were seen. Two of nine patients with osteoid osteoma and nine of 13 patients with osteoblastoma had neurologic disorders before treatment. All patients had open resection of the lesions. Two patients with osteoid osteoma had relapse because of incomplete resection, necessitating a second excision. In 16 of 17 patients with preoperative spinal deformity because of inflammatory effect of the lesion, the deformity improved during followup.
With development of modern imaging techniques, exact surgical planning may become possible; however, in some cases, intraoperative complete resection of the lesion is still difficult.

**Clinical outcome in treatment of parosteal osteosarcoma**

A.H.M. TAMINIAU, P.D.S. DIJKSTRA, H.M. KROON, P.C.W. HOGENDOORN
*(Dep. orthopedic surgery, dep. radiology, dep. pathology. Leiden University Medical Centre, Leiden, The Netherlands)*

Seventeen patients with a parosteal (juxtacortical) osteosarcoma treated between 1980 and 1998 at Leiden University Medical Center were reviewed. The clinicopathological features, diagnosis, surgical treatment, and patient outcome were analysed.
The median age of patients was 35 years, range 13 to 61 years. The distal part of the femur was the most common site (9 patients). The other lesions were located at the proximal femur in 4 patients, at the tibia in 1 patient, at the humerus in 2 patients, and at the radius in 1 patient. A dedifferentiated component was identified in one patient. A wide, marginal and positive surgical margin were found in 9, 4 and 4 patients, respectively. Three patients had a local recurrence treated successfully with further surgery. Surgical staging for stage IA, IB, IIB resulted in 9, 5 and 3 patients, respectively. The different types of reconstruction after resection of the lesion were: an inlay graft in 9 patients, intercalair graft in 3 patients, osteoarticular graft in 3 patients, transposition of the bone in one patient, and no additional reconstruction in one patient. In one third of the patients a local complication established, and no systemic complications occurred. Failure of reconstruction developed in 7 patients, of whom 4 patients with a non-union needed a re-operation. The median functional outcome score (MTSS) for the upper extremity was 24 and for the lower extremity 27.5. Radiological implants system evaluation (ISOLS) for implant and allograft was found to be excellent to good in 11 patients one year after surgery. One of the patients died of the disease (stage IIB) 11 years after surgery. The median follow-up was 6.5 years (range 2.5 to 14.5 years).
Despite failure of reconstructions the functional outcome was good, and the prognosis was excellent.

**Endoprosthesis for metastases to proximal femur**

KHAN M., TILLMANN R., GRIMER R., CARTER S
*(Royal Orthopaedic Hospital Oncology Service. Birmingham, U.K)*

The management of patients with metastatic disease of bone has improved due to advances in chemotherapy and new surgical techniques. The patients are now expected to live longer and demand a better function and improved control of their symptoms.
Metastases to proximal femur are difficult to manage with standard techniques of osteosynthesis as the union may be delayed and implant may fail before fracture healing. Conventional prosthetic replacement has been tried but they are usually associated with problems.
We have used endoprosthetic replacement in sixty-two (62) patients with metastases in proximal femur. The mean age of the patients was 56.7 years ranging from 33–84 years. All patients were followed up till death or failure of the implant. The commonest primary tumours were carcinoma of breast or kidney. Overall survival at twelve months was 68.8% and 27.8% at forty-eight months. All patients reported an improvement in mobility and reduced requirement of analgesia after the endoprosthetic replacement. Dislocation of the prosthesis at the hip joint was the commonest occurring complication. Wound infection was seen in three patients.
We believe that endoprosthetic replacement of the proximal femur offer a successful method of stable reconstruction after resection of the tumour. It has a low incidence of complications and improves the quality of life by reducing pain and facilitating independent mobility.

**Treatment of pathologic proximal femur fractures**

F. PORTABELLA, M. ORDU A, J. CASA AS, D. RODRIGUEZ
*(Orthopaedics Department, Ciutat Sanitaria Bellvitge. Barcelona, Spain)*

*Introduction*: Neoplastic disease treatment by chemotherapy, hormone therapy and radiotherapy has increase the survival of the patients, so bone metastasis has increased. Frequently pathologic proximal fractures femur is a complication in patients outcome.
*Material and methods*: 90 fractures has been surgically treated in the last 7 years. The localization were: head and neck (42) and metaphysis (48). The surgical technique include internal fixation (ender nail 14, screw-plate 4 and screw-plate with cement 12) or arthroplasty reconstruction (Austin Moore 10, Wagner 9, bipolar prosthesis 16, Charnley 8 and Modular prosthesis 17).
*Results*: Recovery extremity function and pain relief was accomplished and functional results by treatment were as follows: ender nail 4 good and 8 poor, screw-plate 4 poor, screw-plate with cement 8 good and 4 poor, arthroplasties 56 good and 6 poor.
The complications were: failure implant 16, infection 3 and prosthesis dislocation 6.
*Conclusions*: Patients with life expectancy more than two months would benefit with surgical treatment. Bone reconstruction with intramedullary nail or screw-plate has been shown to be inadequate at long term because exist a high incidence of failure. Internal fixation augmented with methylmetacrylate to fill the defect and restore the medial cortex has achieved better results. When the tumour extend towards the lesser trochanter we recommended resection and reconstruction with modular prosthesis and when the acetabulum is not involved we tend to use bipolar cups because of their more stability.

**Failure osteosynthesis in the treatment of pathologic intertrochanteric fractures**

F. PORTABELLA, M. ORDU A, J. CASA AS, J. ARMENGOL
*(Orthopaedics Department. Ciutat Sanitaria Bellvitge. Barcelona, Spain)*

*Introduction*: The aim of the study is to evaluate the failure osteosynthesis in the treatment of pathologic intertrochanteric fractures.

*Material and methods*: 56 intertrochanteric fractures has been surgically treated in the last 5 years and we find 20 cases of failure osteosynthesis (13 ender nail, 6 screw-plate, 1 gamma nail). In all the cases the elected surgical procedure was based on the life expectancy estimated by the oncologist.
*Results*: In all the cases had lost of fracture reduction with proximal or distal migration intramedullary nails or the osteosynthesis material became broken. The reasons of failure was the further progression of metastatic disease. The treatment performed were new introduction of nails, arthroplasty joint replacement and medical treatment against pain as needed without other surgical procedure attending the general medical status of this patients.
*Discussion*: The specific plan to treat the pathological intertrochanteric fractures has to take in account several factors: expected survival, type and stage of the tumour, visceral spread, the kind and the extension of bone lesion, etc. One of the more important factor is the life expectancy but in some cases is difficult to precise the survival time, and the surgeon not consider the kind and extension of lesion. The survival of this patients we got failures in the fixation procedures especially when the medial cortex of the femur is not restored. In conclusion we recommend joint replacement by using modular prosthesis in treating intertrochanteric pathologic fractures with large bone destruction affecting patients with an estimated life expectancy higher than six weeks.

**Neo-adjuvant therapy plus conservative surgery in wide soft tissues sarcoma of proximal extremities**

R. CAPANNA, G. BELTRAMI, D.A. CAMPANACCI, M. MUGNAINI, P. DE BIASE, P. OLMI°, M. MELA°, M. PERTICI°
*(Department of Orthopedic Oncology, C.T.O., Firenze ° Department of Radiotherapy, Firenze, Italy)*

In order to achieve a conservative surgery in wide Soft Tissues Sarcoma (STS) of proximal extremities, neo-adjuvant External Beam Radiation Therapy (EBRT) associated to Chemotherapy (CHT) have been introduced in Our Department. We report the experience of six patients, treated from 1998 to 2000, all affected by high grade STS, all with a diameter more than 10 cm. Upper limb (axilla and deltoid region) was involved in two cases and lower (quadriceps or posterior thigh) in four cases. All patients were treated by preoperative chemotherapy (Epirubicin + Ifosfamide, av. 2.5 cycles) plus EBRT (av. 45 GY). After neo-adjuvant treatment, all patients were operated by limb sparing surgery. Wide surgical margins were achieved in all cases. Free flaps (motor unit transfer of innervated latissimus dorsi pro quadriceps) were employed in 2 cases, while rotational flaps were used in one case (motor unit transfer of innervated latissimus dorsi pro deltoid). After surgery, one deep hematoma and one scar slough were observed, surgically repaired. The necrosis, evaluated in the specimens, was excellent in 1 case (100%), good (>90%) in 4 cases and poor in 1 (40%). Adjuvant chemotherapy (Epirubicin + Ifosfamide, av. 2.5 cycles) was employed in all but one cases, while no adjuvant radiation therapy was employed. At an average follow up of 15 months, five patients were continuous disease free, and one patient is alive with multiple pulmonary metastasis. Functional results (according to MSTS) were rated as satisfactory in all cases. In locally advanced STS, neo-adjuvant therapy showed to be efficacy in achieving limb-sparing surgery, by tumoral mass shrinking of proximal extremities. In case of a motor unit transfer, preoperative EBRT is strictly recommended, to avoid damages caused by postoperative EBRT on flaps viability.

**Periosteal Ewing's sarcoma (PES). report of four new cases**

F. DELPINE[2], N. DELPINE[1], G. DELEPINE[1]
*([1] Oncologic Department – Avicenne Hospital – Bobigny; [2] 5 impasse du bon Pasteur, Rouen, France)*

*Introduction*: Few cases of periosteal Ewing's sarcoma have been reported and the surgical implications of such a diagnosis were not underlined. The aim of this study is to evaluate the actual incidence and consequence on surgery.
*Diagnostic criteria*: The reported cases fulfilled the diagnosis criteria defined by Bator:
Ewing's sarcoma of bone histologically confirmed but with a pure periosteal location without medullary extension. In our practice, the computed tomographies proved to be the most reliable exam for diagnosis. Histology of resected specimen may ignore an initial medullary involvement cured by preoperative chemotherapy. On the opposite, M.R.I. can over estimate a PES with intense inflammatory reactive tissue inside the medullary canal.
*Patients*: Out of 132 Ewing's sarcomas of bone of our file, only four (3%) could be classified as PES. All involved the femur in the diaphyseal (2) or metaphysodiaphyseal (2) locations. According to Enneckling classification 2 tumours were graded IIA and 2 IIB. Age of the patient ranged 11 to 18. All patients were treated by resection after preoperative chemotherapy. None was irradiated. With an average follow up of 7½ years, all 4 patients are in first remission.
*Review of the literature*: With these 4 new cases, only 24 cases of PES of bone have been described: 23 out of 24 patients were located on long bones. All were stage II disease. All were treated by chemotherapy and most by surgery (PES seem to have a better prognosis than common form). 22 of 24 patients (91%) are in first remission.
*Discussion*: The surgical incidence of PES must be underlined. When the diagnosis is suspected, then biopsy should be confined to the cortical bone without medullary contamination. Such a procedure permits subsequent partial resection without interruption of the bone continuity, allowing much easier reconstruction.
*Conclusion*: Periosteal Ewing's sarcoma is a rare entity that should be recognized since there is prognosis and surgical incidence.

**Cryosurgery in the treatment of low grade bone sarcoma. preliminary results of six cases**

DELEPINE GÉRARD[1], DELEPINE FABRICE[2], ALKALLAF SALWA[1], DELEPINE NICOLE[1]
*([1] Oncological Ped. Sce Hop. Avicenne France [2] 5 passage du bon pasteur, Rouen, France)*

Introduction: Cryosurgery has been used extensively for many aggressive or recurrent benign tumors but till now few cases were described in treatment of bone sarcomas.
*Patients*: Five patients (4 males and 1 female aged 17–56 years) with low grade bone sarcomas were treated by curettage and cryotherapy between March 1996 and July 2000. Histology was chondrosarcoma in 4 and multifocal epithelioid hemangioendothelioma in one. As the patient with epithelioid hemangioendothelioma received cryotherapy for 2 locations, 6 different tumors were treated: 2 proximal tibia, 2 distal femur, 1 os calci and one sacral bone. Cryotherapy was preferred to classical treatment by wide resection to avoid amputation in 2 patients, extra-articular resection in 1, massive prosthesis in 1 and major neurologic sawquellae in 1.
*Method*: Following intra-capsular curettage the residual cavity was filled with liquid nitrogen. The freeze was maintained for several minutes and then allowed to thaw. Freezing and thawing was repeated 2 or 3 times. In all the limb locations the cavity was then filled with methacrylate and osteosynthesis performed to prevent secondary fracture. After operation immediate mobilization and full weight bearing were authorized. On August 1, 2000, median follow up is 36 months.
*Results*: 2 skin sloughs (one followed by deep infection of os calci) were observed and compelled to reoperate. The patient with a chondrosarcoma of the sacrum suffered from painful paresthesia during 6 months. No fracture nor recurrence was observed till now. According to the EMSOS criteria, the late functional score was rated excellent in 3 patients and good in 2.
*Conclusion*: In selected low grade bone sarcoma, cryotherapy yields oncologic results as good as marginal resection and permits much more conservative surgery. Despite impredictability and complications of freezing, cryosurgery should be considered for low grade intracompartimental (grade IA) bone sarcoma. More cases and longer follow up are needed for definitive conclusions.

**Parosteal osteosarcoma of the tarsus with vertebral metastasis: a case report**

FERNÁNDEZ SEARA P, ECHEGOYEN A, LÓPEZ COUSILLAS A, REPÁRAZ B
*(Dpt. of Pathology. Virgen del Camino Hospital. Pamplona. Spain)*

*Case report*: A 49-year-old man who 6 years ago suffered a sprain in the right ankle, and since then discomfort in the tarsus. In that moment, a radiograph showed a hyperdense exofitic lesion in the tarsal scaphoid that was diagnosed as hyperdense exostosis. Two years later the discomfort persisted, and the radiograph showed an increase in size. Four years later the patient was examined because of an increase of the pain, and roentgenographically the lesion had grown with an aggressive pattern, osteocondensed, suspicious of malignancy. A biopsy was performed and the diagnosis was of Parosteal Osteosarcoma (PO). At the time of the biopsy the patient developed back pain and the TAC revealed a destructive osteocondensed lesion in T8; the biopsy was identical to the tarsal lesion: PO.
*Discussion*: PO is a very rare neoplasm, accounting for less than 1% of malignant bone tumors (7). The most common locations are femur, tibia or humerus; the tarsal bones are unusual locations. The pathologic diagnosis is very difficult, because they are well-differentiated osteogenic tumors with only slight cytologic atypia, and for this reason are required clinical and roentgenogram references; the differential diagnosis may include diverse benign entities such as Fibrous Dysplasia, Osteocartilaginous Exostosis, Parosteal Osteochondromatous Proliferations, Ossificans Myositis, etc. The treatment is conservative surgery, and the prognosis is very good but metastases have been described in very unusual occasions. Another special feature of this case is the synchronous diagnosis of the two tumors with identical histopathologic picture. Synchronous bone tumors have been described in the literature, but in this case we think the scaphoid lesion is a primary parosteal sarcoma causing metastases in T8 vertebral body after 6 years of natural development, revealing the potentially but unusual malignant behavior of this neoplasm.
*Conclusions*: PO is a very rare neoplasm among bone tumors. Tarsal location is unusual. The prognosis is usually very good, but we must remember its malignant biological nature, like this case.

**Osteoid osteoma treated by percutaneous radiofrequency ablation: the C.T.O.–florence experience**

CAPANNA R*, MICHELIS B*, CALDORA P*, CAMPANACCI D A*, BELTRAMI G*, MUGNAINI M*, MAZZA E°
*(Department of Orthopaedic Oncology. C.T.O. ° Department of Radiology; Careggi Hospital, Florence, Italy)*

Percutaneous radiofrequency coagulation has become a highly successful alternative to surgical treatment of the osteoid osteoma (o.o.) using long wavelength electromagnetic radiation to produce thermal ablation.
*Materials and methods*: From 1998, 19 patients underwent percutaneous radiofrequency ablation for o.o. in our Institution: 9 men and 10 women, with an average age of 28 years; 18 o.o. were located in the limbs and 1 in D7 vertebral arch. Under CT guidance a Kirschner wire was positioned inside the nidus with a hand drill. An hollow needle biopsy (8 gauge) was subsequently advanced over the K wire and then utilised as a guide for the thermocoagulating probe after removing the K wire. By using a 1 cm tip length probe, attention should be taken to the final position (confirmed by CT scan) since no more than 5 mm of tissue around the probe will be burned. In benign tumors bigger than 1 cm, a 2 cm tip probe may be used or, in alternative, multiple coagulation will be necessary. In our experience a Radionics Cool-tip RF Generator System has been used in all the patients. The electrode is gradually brought to 90 degrees C° working for 6 minutes on average. Patients have been evaluated considering the pain relief and control CT examinations were performed immediately following the procedure and after 1, 6, 12 and 24 months.
*Results*: At an average follow up of twelve months (minimum 3 max 24) the procedure has been considered successful in 17 patients (90%). In 2 cases there was no pain relief: 1 because an imaging simulating an osteoid osteoma and 1 not responding to ablation and undergone to surgical resection. Two minimal complications were observed: both consisting in skin-burns. Hospitalisation time has been of 24 hours in all cases.
*Conclusion*: Percutaneous thermocoagulation of the o.o. results to be an effective, simple and minimally invasive technique in alternative to the traditional surgical approach. This procedure is particularly indicated in osteoid osteoma deeply located, requiring an aggressive surgical approach and for vertebral locations. In our experience this procedure was successful in 90% of cases with no major complications.

**Management of giant cell tumours of the distal radius**

KHAN M., GRAY J., CARTER S., GRIMER R., TILLMAN R
*(Royal Orthopaedic Hospital Oncology Service. Birmingham, U.K)*

Giant Cell Tumours of the distal radius are rare. Curettage has been reported to be associated with high incidence of recurrence. Twenty-three patients were referred to our unit over a 28 year period with a distal radius Giant Cell Tumour, were graded according to the Campanacci system and underwent meticulous curettage. Four patients developed recurrence, one of grade I, and three grade III. Single curettage alone eliminated tumour in 19 patients (82.6%) and 22 (95.7%) with a second curettage. Complications occurred in 4 patients, all of which were grade III. Carpal tunnel syndrome and ulnar impingement occurred in one and two patients respectively, and required further surgery. One patient underwent below-elbow amputation following repeated curettage of a grade III tumour. Single curettage was curative in 17 of 18 patients (94.4%) with grade I or II tumours. Higher rates of complications and recurrence occur in grade III tumours but meticulous curettage of early lesions offers the best chance of cure.

**Reconstruction surgery in bone tumours, limb salvage surgery using autoclaved bone graft.**

N. A. RIBEIRO, J. B. SALGUEIRO, J. S. AMARAL.
*(Egas Moniz Hospital, Lisbon, Portugal.)*

The authors have done a retrospective clinical and radiological study of four patients with malignant bone tumours treated by wide margin resection of the tumour, followed with reimplantation of the resected autoclaved bone.
There was a male and three female patients with an average age of 36.5 years (aged between 15–51 years). Histologically, three were chondrosarcoma and the fourth a Ewing sarcoma. Resected length of the proximal femur (two patients), distal femur (one patient) and distal tibia (one patient), ranged from 10 to 23 cm. After cartilage and soft tissue removal, the resected segment was autoclaved for 8 minutes at 132°C. A 0.2 megaPascal pressure was used. None of the tumours recurred and at the most recent follow-up (from 12 to 121 months with an average follow-up of 46.7 months), all the patients were disease free. All the patients were available for radiological assessment and evident bone union was seen in three cases. This method of reconstruction using autoclaved bone graft has some advantages and can be useful in large bone resections, along with prosthetic or other implants. It seems to be a valid alternative to graft from bone bank, particularly in areas where complex bone morphology (proximal femur, for instance) makes perfect anatomical rearrangement with allograft difficult to achieve.

**Shoulder surgery in malignant bone tumours**

JO O B. SALGUEIRO, JORGE E. LOPES, NUNO A. RIBEIRO, RUI G. GORDO
*(IPOFC de Lisboa/Hospital de Egas Moniz, 1990–2000)*

The authors have done a retrospective clinical and radiological study of seven patients (four females and three males) with shoulder malignant bone tumours operated between 1990 and 2000.
The median age was 43.3 years (17–59 years) and the median disease free interval was almost 5 years (5–137 months).
In four cases the histological diagnosis was chondrosarcoma (umerus – 3 patients, scapula – 1 patient), in one case mesenquimal desdiferenciated tumour (scapula), and osteosarcoma (umerus) and a Ewing sarcoma (scapula) in the other two cases.
The authors discuss the functional results, the morbility of the procedures, and the mortality.

### ABSTRACTS FOR NURSES SYMPOSIUM

**P.A.E. in children and adolscents with cancer experiencing nutritional problems in patient and out patient protocol**

E. MARIN, D. COLUNGA
*(Pediatric Ward, Clinica Universitaria, University of Navarra. Pamplona, Spain)*

*Introduction*: In recent years there have been significant advances made in the treatment of cancer in children and adolescents. These have improved survival rates but have also increased secondary effects. From the nursing point of view and the experience of our Paediatrics Department, we have found a significant deterioration in the nutritional state of children and adolescents not only at the diagnostic stage but also during treatment.
The occurrence of weight loss, nausea and vomiting, aversion to food, alteration gustatory, electrolyte have prompted us to consider the need for a nursing protocol for in-patients and out-patients established on the basis of a through assessment of the nutritional-metabolical and elimination patterns in the child and adolescent.
*Aims*: To detect the most common nutritional problems during treatment.
To establish a nursing care plan related to diagnosis.
To formulate a protocol for in-patients and out-patients.
*Material and methods*: Descriptive and retrospective study of nursing documentation in the light of the nutritional-metabolical and elimination patterns established by M. Gordon and of the problems related to nursing diagnosis associated with these patterns.
Our experience in terms of the assessment of continued nursing care during diagnosis and treatment.
*Discussion and conclusions*: The need to formulate a protocol to counter the nutritional and metabolical deficiencies in children and adolescents as well as the problems related to elimination

**Specific issues of children in hospital**

G. CUGINO, M. MERCURI, M. MANFRINI
*(5[a] Divisione Chirurgica Oncologica – Istituto Ortopedicao Rizzoli. Bologna, Italy)*

The surgical ward of the Department of Musculoskeletal Oncology at the Istituti Ortopedici Rizzoli specializes in the surgery of neoplasms of bone and soft tissues. Nurses of this ward work with patients of very different ages. During the years 1998–99, 247 patients aged less than 14 years and 1591 adult patients were admitted in this ward. Among the 247 pediatric patients, 48 underwent major surgery (rotationplasties, vascularized graft, etc).
The aim of this study is to focus on the main problems of treating and communicating with pediatric patients, according to:

– specific issues related to patients' specificity:
- age and development: the ability of understanding the current situation and the context vary in relation to the age of the children, so staff's explanations and behaviour must be chosen accordingly;
- children behaviours: children have to move in a safe environment and the nursing-staff must be responsible for that;
- information: nurses must know the specific ways to interact effectively with very young patients;
- employment of time: the possibilities to play, to move, and to study are essential for these patients; parents may be involved in these activities;
- stress: the disease, the therapy and the clinical tests (blood tests, CT scans, MRI, scintigraphy, etc.) together with the interruption of normal life, all contribute to a very high level of stress, which has to be controlled;
- pain.

– steps of diagnosis/treatment:
- hospitalization
- preparation to diagnostic procedures
- pre-operative preparation
- medication and plaster devices

**The protocolization of nursing care as a measure to diminish complications in the use of "implanted port"**

M. REMIRO, P. MIGUELEIZ, S. PÉREZ
*(Pediatric ward. Clinica Universitaria, University of Navarra. Pamplona, Spain)*

Since year 1983, the Paediatric department of the C.U.N. used the "implanted port" as a means of reaching the venous tract in children with onco-hematologic diseases.
By studying 260 implanted ports in our Paediatric unity, as well as revising the bibliography, we have found a series of complications derived from the use of this system, principally obstructions and infections.

A protocol of nursing activities was elaborated based on a comparative study. The application of this protocol allowed diminishing the complications.
The comparative study and the protocol are presented with the purpose of demonstrating its effectiveness.

**Postoperative pain control with infusor in oncologic patients**

V. DELFINO, P. BONUCCELLI, F. ANEDDA, M. CARAVELLA, A. CORAL, C. GRASSELLI, M. MARTINI, N. MORRONE, C. NERI, G. PRINCIGALLI, S. ROSADI, R. SANTONI, G. SANTANGELO, C. SCIASCIA, F. TANGANELLI, S. VICIANI
*(Department of orthopaedic oncology and reconstructive surgery C.T.O. Fiorence, Italy)*

The aim of the study was the evaluation of the efficacy of analgesic drug continuous delivery by an infusor (elastomeric pump) for postoperative pain control in patients undergoing surgical procedures for bone or soft tissue tumours. From May to December 2000, were recruited 100 patients: (47 females and 53 males) ranging in age from 16 to 80 years; 11 patients underwent amputation, 89 were treated by resection or excision. Limb salvage surgery was done in 43 soft tissue sarcoma and 46 bone tumours. These patients were monitored by the nurses' team following two different protocols; a) analgesia by epidural infusor, b) analgesia by intravenous infusor. Drugs used by peridural infusion were Morphine 20 mg, Marcaina 0.5% 50 ml. physiologic solution 50 ml. Drugs used by intravenous infusion were Morphine 20 mg. Ketorolac trometamina 120 mg. Physiologic-solution 90 ml. The level of analgesia was monitored every 3 hours along 48 hours after surgery using the VAS score system. The efficacy of the analgesia was judged considering the amount of additional FANS somministration: GOOD (30 mg/48 hours/ketorolac trometamina) FAIR (60 mg/48 hours/ketorolac trometamina) POOR (>60 mg/48 hours/ketorolac trometamina). The pain control resulted as GOOD in 83% of patients, FAIR in 11% and POOR in 6% cases. No major adverse reaction and no local or general complications related to the analgesic treatment occurred (infections, tromboflebity, etc). Respect to i.v. administration drugs delivery by peridural catheter showed major disadvantages: a higher risk of incorrect insertion or subsequent displacement of the catheter particularly in case of excited patients or having several additional sources of pain (as multiple metastases, respiratory distress, etc.). The use of infusor (either i.v. or peridural) showed several advantages: 1) professional autonomy of the nurse team. 2) rational use and choice of the different drugs. 3) simple technique to perform and to maintain. 4) easy monitoring of the level and continuity of the analgesia. 5) excellent compliance by the patients. In conclusion, the present protocol showed in most of cases an excellent postoperative pain control, improving also the psychological condition of the patients who feel himself constantly cared, and followed.

**Nursing psychological care to parents of children hospitalised with cancer**

A.B. MU OZ, A. SARSOLA, A. TREVI O, S. LAFUENTE
*(Pediatric Ward. University Clinic, University of Navarra. Pamplona, Spain)*

Research reveals that after diagnosis, hospitalisation is the second most stressful event for families having a child with a chronic condition.
Teamwork with an interdisciplinary approach has been found to be necessary in order to meet patients and their families needs correctly.
Starting from this point, the review of the literature and the analysis of the current situation of our patients and families, the nursing team from the paediatric war in the University Clinic of Navarre has developed a group of activities. With this protocol we pretend to help parents to face the illness and hospitalisation of their children, and in this was to improve, promote and achieve their adaptation to this situation.

**Can a relative "become" a good health worker in managing the central venous catheter of the tumor patient? experience of the rizzoli orthopaedic institute (bologna-italia)**

C. FORNI, L. LORO, T. MAZZEI, C. BERGHELLI, A. BIOLCHINI, M. TREMOSINI, A. TREGGIANI, C. RASPANTI
*(Department of musculo-skeletal tumors – Rizzoli Orthopaedic Institute. Bologna, Italy)*

The chemotherapy unit for bone tumours is a ward of 12 beds in the department of musculo-skeletal tumours in the Rizzoli Orthopaedic Institute – Bologna, Italy. Only 5% of the patients in the center come from our region, the rest are from other regions or abroad. These patients are affected by osteosarcoma or Ewing's sarcoma of the extremities. In 80% of cases a long-term central venous catheter (CVC) is inserted not only to carry out cycles of chemotherapy in the hospital but also to perform repeated blood tests, intravenous therapies and transfusions needed at home. To ensure that treatment is really effective and at the same time safe, the parents of each patient are taught all the procedures to manage the CVC at home. This is to enable blood to be taken, and infusion, medication and heparinisation to be carried out. This is advantageous compared to putting these patients in the hands of the family doctor or peripheral health structures that are often not competent in the management of these devices. The nursing team, backed up by doctors, have designed their own training course to acquire the necessary know-how in order to teach people without specific skills some complex healthcare procedures. Subsequently, a training course was set up to relatives, which consists of a theoretical part and practical one. For theory written and audio-visual material is used; for the practical lessons a small laboratory has been equipped with a professional dummy and the material needed for simulations. 52 patients entered in the study between June 1999 and June 2000 infection rate decreased from 10.1% to 7.8% malfunction from 43% to 23% and only 1.6% never used the CVC at home compared to 7.5% the year before. The analysis of results shows that the aims have been fulfilled.

**A support group for young adult survivors of childhood musculoskeletal cancer: concerns and dilemmas**

L. BEN-AMI, R. BERTY, O. ARBEL, E. SINGER, I. MELLER, P. ELAD
*(Departments of Social Services, Psychiatry and Orthopedic Oncology. Tel Aviv Sourasky Medical Center – Tel Aviv, Israel)*

Nine young adult survivors of childhood musculoskeletal cancer participated in a support group held in Tel Aviv Sourasky Medical Center. Their age range was 19–25 years, and the average length of time since diagnosis was six years. The group met once monthly over a period of two years. The department's social worker and psychologist led the group. The head of the department and head nurse also participated in the meetings. The aim of the support group was to investigate how the diagnosis of cancer in the past affected present day functioning. Emphasis was placed on aspects relevant to the stage in the participant's life cycle such as relationships with members of the opposite sex and choice of occupation. A central issue in the group discussion was how much being a survivor of childhood cancer becomes part of a person's identity. Another concern was the connection between personal acceptance of any existing disability and acceptance of it by others. The fact that three group participants were young Arab women helped facilitate a discussion of cultural differences in coping and adjustment. The participants reported that the meetings helped them in accepting their past trauma and in learning appropriate coping styles of behaviour of their current status. Our experience indicates that young adult cancer survivors can benefit from a support group, that focuses on their current needs.

**Centralized cytotoxic unit in a pharmacy service**

T. GARCIANDIA, GARAYOA N, RUIZ DE LAS HERAS R, URDANOZ M[A] JOSÉ, ELCARTE B.E
*(Pharmacy Service. Clínica Universitaria. University of Navarra. Pamplona, Spain)*

The use of antineoplastic drugs has increased during the last years as well as the awareness of the potential risk associated with their manipulation, preparation and administration.

As a consequence, some scientific societies have developed guidelines to motivate the correct manipulation of these drugs.

Since the beginning of cytotoxic therapy Hospital Pharmacy Services, as those responsible for the correct use of drugs, have pursued the preparation of cytotoxics in centralized units. Their objectives with these units have been to protect to be manipulated (aseptic manipulation of the drug) as well as the manipulator (adequate facilities and working techniques).

In this presentation we describe the main characteristics that cytotoxic centralized units of Pharmacy Services must have, measures that are available for individual protection and working techniques.

Regarding facilities it is essential to have an area with restricted access, in which there must be a biological safety cabinet vented to the outside and with negative pressure.

Individual protection kit includes impermeable gowns, mask and thick gloves.

In order to obtain the maximum benefit from the above measures it is important to have personnel adequately trained in: antineoplastic drugs, working procedures in laminar airflow cabins, reconstitution and dilution techniques, and the use of needles with de spike filters (0.22 micres) or other mechanisms for aerosol avoidance.

In summary, Pharmacy Services have tried to warrantee the safety of the patient (correct drug and dose, most convenient dilution, sterile preparations) and that of the cytotoxic manipulator (use of laminar airflow cabins, equipment for personnel protection, and adequate training).

## References

[PDF_00220] Andreasen P. A., Kjøller L., Christensen L., Duffy M. J. (1997). The urokinase-type plasminogen activator system in cancer metastasis: a review.. Int J Cancer.

[PDF_00219] Campanacci M., Bagnara G. P., Serra M., Giovannini M., Tomasi P., Pileri S., Poggi S., Lollini P. L., Picci P., Paolucci G. (1989). Giant cell tumor of bone: a model for the in vitro human osteoclast characterization.. Tumori.

